# Coronavirus disease-related in-hospital mortality: a cohort study in a private healthcare network in Brazil

**DOI:** 10.1038/s41598-022-10343-4

**Published:** 2022-04-16

**Authors:** Helidea de Oliveira Lima, Leopoldo Muniz da Silva, Arthur de Campos Vieira Abib, Leandro Reis Tavares, Daniel Wagner de Castro Lima Santos, Ana Claudia Lopes Fernandes de Araújo, Laise Pereira Moreira, Saullo Queiroz Silveira, Vanessa de Melo Silva Torres, Deborah Simões, Ramiro Arellano, Anthony M.-H. Ho, Glenio B. Mizubuti

**Affiliations:** 1 Rede D’Or, Av. Presidente Juscelino Kubitschek, 1839, São Paulo, SP Brazil; 2 Department of Anesthesiology, São Luiz Hospital-Rede D’Or-CMA, Rua Alceu de Campos Rodrigues, 229, São Paulo, SP Brazil; 3 Department of Infectious Disease, São Luiz Jabaquara Hospital-Rede D’Or, Rua Perobas, 344, São Paulo, SP Brazil; 4 Department of Anesthesiology and Perioperative Medicine, Queen’s University, Kingston Health Sciences Centre, Kingston General Hospital, 76 Stuart Street, Kingston, ON K7L 2V7 Canada

**Keywords:** Respiratory tract diseases, Health care

## Abstract

COVID-19-related in-hospital mortality has been reported at 30.7–47.3% in Brazil, however studies assessing exclusively private hospitals are lacking. This is important because of significant differences existing between the Brazilian private and public healthcare systems. We aimed to determine the COVID-19-related in-hospital mortality and associated risk factors in a Brazilian private network from March/2020 to March/2021. Data were extracted from institutional database and analyzed using Cox regression model. Length of hospitalization and death-related factors were modeled based on available independent variables. In total, 38,937 COVID-19 patients were hospitalized of whom 3058 (7.8%) died. Admission to the intensive care unit occurred in 62.5% of cases, and 11.5% and 3.8% required mechanical ventilation (MV) and renal replacement therapy (RRT), respectively. In the adjusted model, age ≥ 61 years-old, comorbidities, and the need for MV and/or RRT were significantly associated with increased mortality (p < 0.05). Obesity and hypertension were associated with the need for MV and RRT (p < 0.05).

## Introduction

One of the significant concerns in the course of the COVID-19 pandemic is the existence of high-risk groups^1^. Indeed, research has shown a higher incidence of disease progression to severe lung compromise in older men with comorbidities^2^. Yet, despite the rapid rise in the COVID-19 literature since the beginning of the pandemic (at the time of writing, a PubMed search revealed > 237,000 related publications), studies are still needed to further elucidate its epidemiological characteristics and identify risk factors associated with poor outcomes in different populations^3^.

Brazil has a large, heterogeneous and unevenly distributed population, with nearly 90% of its ~ 210 million inhabitants concentrated in the Northeast, Southeast and South regions, which together account for only ~ 1/3 of the country’s territory. Furthermore, its continental dimensions invariably bring significant cultural and climatic disparities. Also (and perhaps most) noteworthy, important socioeconomic inequalities can be exemplified by major disparities in access to (and in quality of) healthcare, particularly between the private (to which only ~ 1/4 of the population has access, with a likely predominance of younger individuals who are part of the Brazilian workforce) and the public healthcare systems^2,4,5^. Nevertheless, COVID-19 is widely distributed across Brazil’s 5 macro-regions (North, Northeast, Central-West, Southeast, and South) with not only a high incidence, but also a high, albeit uneven, in-hospital mortality, which likely relates in part to the existing regional discrepancies of the country’s healthcare system^4^. Previous studies, which combined account for ≥ 1 million patients hospitalized due to COVID-19 in Brazil, have reported mortality rates ranging from 21.7 to 47.3%^4,6–10^. All of these studies, however, originated from “SIVEP-Gripe”—the main Brazilian Ministry of Health’s (BMH) repository of notifications of COVID-19 hospitalizations—and none of them discriminated the in-hospital mortality between public and private institutions^4,6–10^. Despite high heterogeneity between (and within) the Brazilian public and private health systems, public hospitals have been largely and chronically underfunded compared to the private sector which (indisputably) has overall better infrastructure and offers better remuneration to healthcare personnel (thereby attracting the most qualified human resources). It is therefore plausible that the COVID-19-related in-hospital mortality would differ between these two sectors. To date, however, no studies have examined the in-hospital mortality (and associated risk factors) in a large cohort of COVID-19 patients admitted exclusively to private Brazilian hospitals. Importantly, indentifying risk factors independently associated with poor outcomes is of major interest as it allows governments and other stakeholders to tailor health actions (e.g., allocation and/or creation of hospital beds, distribution of human resources) according to the local population risk profile. We therefore sought to determine the in-hospital COVID-19-related mortality, and to examine the correlation between (i) demographic variables, (ii) presence and (iii) number of comorbidities, and need for (iv) mechanical ventilation (MV) and/or (v) renal replacement therapy (RRT) with the occurrence of in-hospital death in a private Brazilian healthcare network. Notably, despite recent literature linking individual’s genetic profiles with COVID-19 susceptibility and severity^11^, this is out of the scope of the present investigation.

## Results

**Figure 1:**
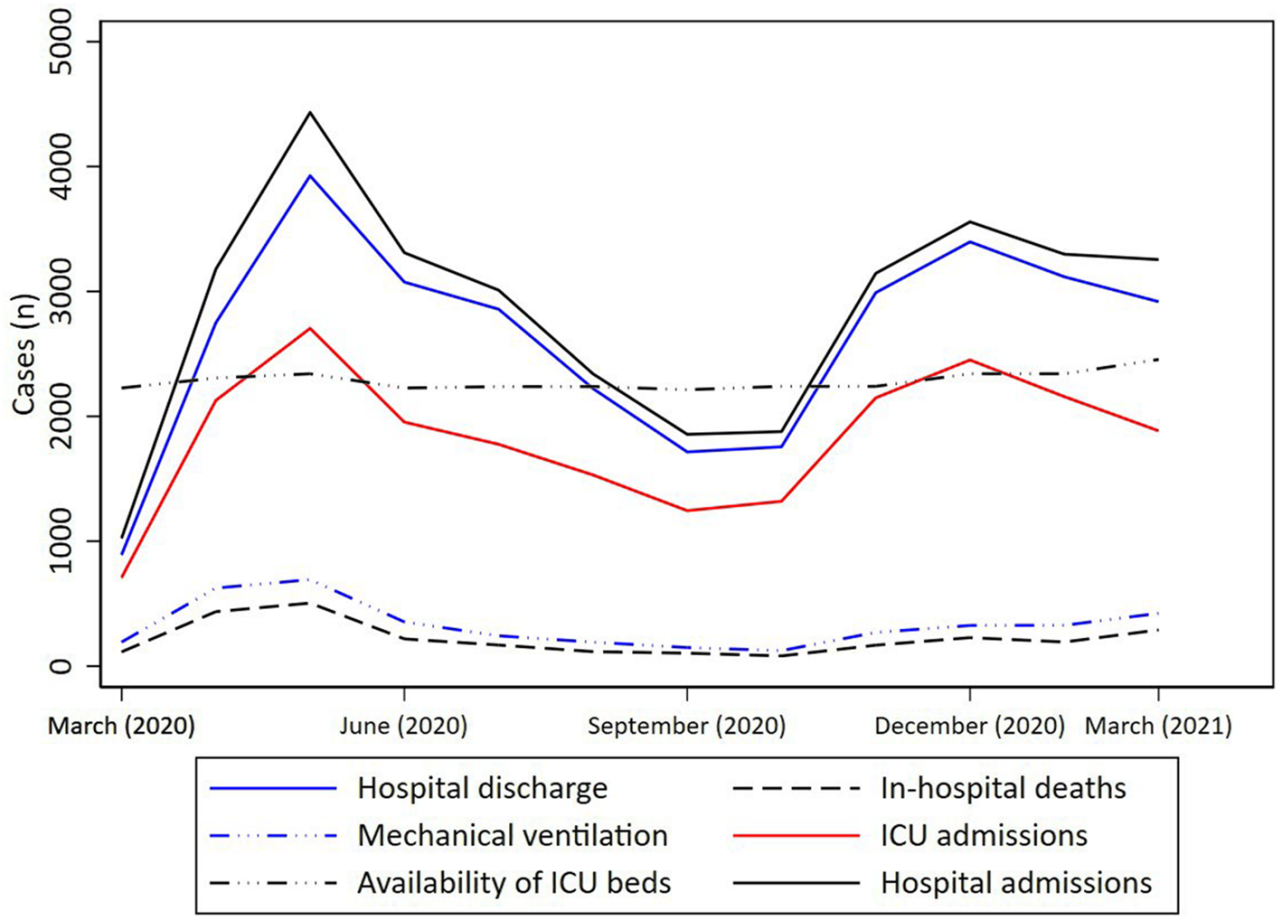
Patient distribution according to number of hospital admissions, hospital discharges, in-hospital deaths, patients on mechanical ventilation, intensive care unit (ICU) admissions, and the variation on ICU-bed availability from March 1st, 2020 to March 31st, 2021.

In total, 38,937 patients were hospitalized and 3058 (7.8%) died of COVID-19 (Supplementary Tables 1 and 2). The mean age of the hospitalized patients was 53.2 ± 17.0 years. 57% of the patients were men. 74.0% were from the Southeast region, 17.9% from the Northeast, 5.9% from the Central-West, and 2.2% from the South (Supplementary Fig. 1). Patients who died had a higher mean age than those discharged from hospital (p < 0.001) (Supplementary Fig. 2). Figure 1 shows the number of hospital admissions, hospital discharges, in-hospital deaths, patients on MV, intensive care unit (ICU) admissions, and the variation of ICU-bed availability during the studied period. Also, we compared the ICU-bed availability between a pre-pandemic period (i.e., from October, 2019 to January, 2020—1733 ICU beds available) with the peak (May, 2020) of hospital admissions due to COVID-19 (Fig. 1). In total, 607 new ICU beds were created corresponding to a 35% increase in ICU-bed availability compared to the pre-pandemic period. There was no correlation between ICU admission and in-hospital mortality (rho = 0.31, *t* = 1.11, p = 0.28), whereas a strong correlation was observed between the need for MV and in-hospital death (rho = 0.97, *t* = 15.5, p < 0.001) (Supplementary Fig. 3).

The risk of death increased with increasing age, starting at 41 years-old (p < 0.05), and peaking at ≥ 81 years-old (HR = 7.76; p < 0.001) (Table 1). There was no relationship between sex and in-hospital mortality (Table 1). The mean hospital length of stay (LOS) was 10.0 ± 8.2 days (9.3 ± 7.4 days for patients discharged from hospital and 18.1 ± 11.9 for patients who died; p < 0.001).

**Table 1:** Demographic variables of hospitalized patients with coronavirus 2019 disease (from March 1st, 2020 to March 31st, 2021). Unadjusted analysis using the Cox regression model.

Characteristics	Unadjusted univariate analysis				
	Discharge % (n)	Death % (n)	HR	CI (95%)	p-value
Total	92.2 (35,879)	7.8 (3058)	–	–	–
Age (years)^a^	51.6 ± 16.2	71.6 ± 15.6	1.3	1.03–1.39	< 0.001
**Age groups (years)**^**b**^					
20–30	99.1 (2624)	0.9 (24)	1.00		
31–40	98.4 (7677)	1.5 (121)	1.32	0.85–2.06	0.20
41–50	97.6 (8415)	2.4 (209)	1.69	1.11–2.59	0.01
51–60	95.9 (7113)	4.1 (306)	2.04	1.35–3.10	0.001
61–70	88.5 (4861)	11.5 (630)	3.96	2.63–5.96	< 0.001
71–80	80.7 (3126)	19.3 (745)	5.34	3.55–8.03	< 0.001
≥ 81	66.9 (2063)	33.1 (1022)	7.76	5.17–11.65	< 0.001
**Sex**^**b**^					
Female	92.6 (15,504)	7.4 (1234)	1.00		
Male	91.8 (20,375)	8.2 (1824)	0.95	0.89–1.03	0.25
**Geographic distribution**^**b**^					
Central-West	92.6 (2111)	7.4 (169)	1.00		
Northeast	87.5 (6085)	12.5 (872)	0.88	0.74–1.04	0.14
Southeast	93.5 (26,935)	6.5 (1880)	0.66	0.56–0.77	< 0.001
South	84.5 (748)	15.5 (137)	1.74	1.38–2.19	< 0.001

*HR* Hazard ratio, *CI* confidence intervals.

^a^Values are expressed as means ± standard deviations. ^b^Values are expressed as relative and absolute frequencies.

The unadjusted analysis of comorbidities showed that all independent variables were significantly associated with increased mortality. The median (25–75% percentile) number of comorbidities was 2 (2–3) for patients who died and 1 (0–2) for those discharged from hospital (p < 0.05) (Table 2).

**Table 2:** Distribution of hospital discharges and deaths according to presence and number of comorbidities and the need for mechanical ventilation and renal replacement therapy in patients hospitalized due to coronavirus 2019 disease (from March 1st, 2020 to March 31st, 2021). Unadjusted analysis using the Cox regression model.

Comorbidity*	Unadjusted univariate analysis				
	Discharge % (n)	Death % (n)	HR	CI (95%)	p-value
Chronic obstructive pulmonary disease^a^	67.6 (696)	32.4 (334)	2.08	1.85–2.34	< 0.001
Asthma^a^	94.0 (1632)	6.0 (104)	0.82	0.67–1.00	0.055
Cardiovascular disease^a^	77.4 (2470)	22.6 (719)	1.76	1.61–1.91	< 0.001
Cerebrovascular disease^a^	64.1 (596)	35.9 (334)	2.34	2.08–2.63	< 0.001
Hypertension^a^	86.9 (12,536)	13.1 (1883)	1.54	1.43–1.66	< 0.001
Diabetes mellitus^a^	83.9 (6703)	16.1 (1290)	1.63	1.52–1.76	< 0.001
Obesity (BMI > 30 kg/m^2^)^a^	92.3 (7460)	7.7 (621)	0.89	0.81–0.97	0.01
Chronic kidney disease^a^	56.8 (678)	43.2 (516)	2.37	2.14–2.61	< 0.001
Immunosuppression^a^	69.7 (573)	30.3 (249)	2.16	1.89–2.46	< 0.001
**Number of comorbidities**^**a**^					
0	98.5 (11,550)	1.5 (181)	1.00		
1	95.3 (11,239)	4.7 (548)	2.02	1.71–2.42	< 0.001
2	89.7 (7337)	10.3 (840)	3.09	2.62–3.65	< 0.001
3	84.2 (3857)	15.8 (723)	3.77	3.19–4.46	< 0.001
≥ 4	71.2 (1893)	28.8 (766)	5.23	4.43–6.19	< 0.001
Need for mechanical ventilation^a^	40.4 (1803)	59.6 (2664)	14.86	13.28–16.64	< 0.001
Need for renal replacement therapy^a^	33.5 (493)	66.5 (980)	3.71	3.40–4.05	< 0.001

*HR* Hazard ratio, *BMI* body mass index, *CI* confidence intervals.

*Information obtained from the patient and/or accompanying members through the initial anamnesis questionnaire during hospital admission.

^a^Values are expressed as relative and absolute frequencies.

Admission to the ICU occurred in 62.5% (n = 24,336) of the cases, of whom 9.1% (n = 2209) died. Notably, out of the observed 3058 in-hospital deaths, 2209 (72.2%) occurred in the ICU, 820 (26.8%) in the step-down unit, and 29 (0.9%) in the ward (Supplementary Fig. 4). The mean ICU LOS was 8.8 ± 8.7 days. MV was instituted in 4467 patients, corresponding to 11.5% of our entire cohort and 18.3% of the patients admitted to the ICU. All patients requiring MV were ventilated in the ICU. The mean time of MV was 12.4 ± 13.0 days (11.3 ± 11.6 for patients who were discharged and 13.2 ± 13.9 for those who died; p < 0.001). Of the patients who required MV, 14.8% (n = 656) remained mechanically ventilated for > 21 days. RRT was instituted in 3.9% (n = 1473) of patients. Of the patients on MV, 28% (n = 1253) required RRT. In the unadjusted analysis, the variables selected as surrogates for progression of disease severity (namely, need for MV and/or RRT) were significantly associated with in-hospital mortality (Table 2). Extracorporeal membrane oxygenation was implemented in 79 patients (0.2%), of whom 43 (54.4%) died. Specific analyses correlating these patients’ demographics, comorbidities, duration of hospitalization, and indicators of progression of disease severity (i.e., need for MV and/or RRT) with the study outcomes (i.e., hospital discharge or in-hospital death) can be found in Supplementary Figs. 5 and 6, and Supplementary Tables 3–5.

**Figure 2:**
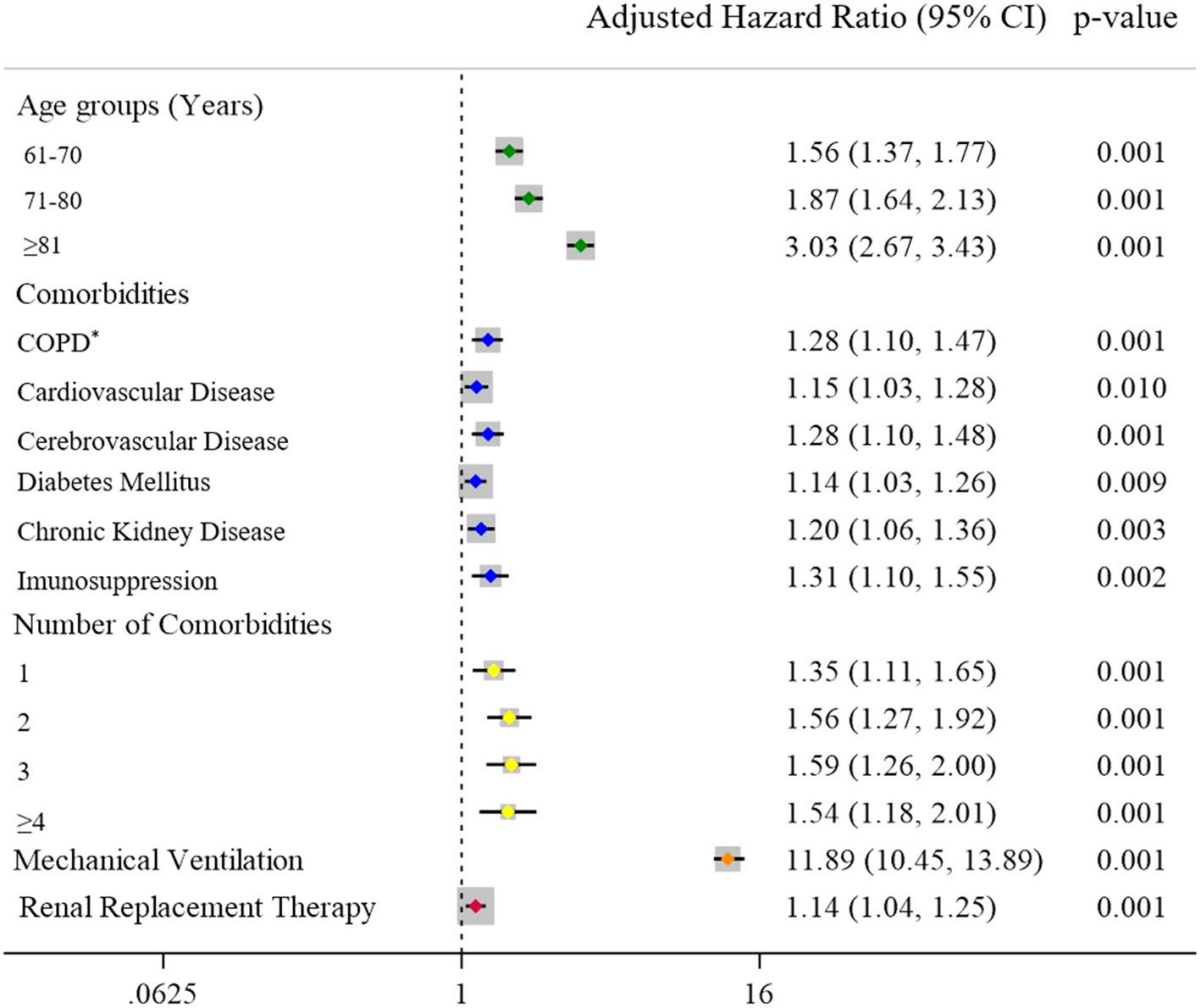
Variables associated with in-hospital mortality in patients hospitalized due to coronavirus 2019 disease between March 1st, 2020 and March 31st, 2021. Analysis adjusted by the Cox regression model. *Chronic obstructive pulmonary disease.

In the adjusted analysis, (i) age > 61 years-old, (ii) presence and (iii) number of comorbidities, and (iv) need for MV and/or (v) RRT remained significantly associated with in-hospital mortality (Fig. 2 and Supplementary Fig. 7), with age ≥ 81 years-old and need for MV being the most prominent risk factors (Supplementary Fig. 8). In-hospital mortality stratified by age range and (i) number of comorbidities, (ii) need for MV, and (iii) need for RRT can be found in Supplementary Tables 6–8. The best fitted model was the one with age, comorbidities, number of comorbidities, the need for MV and RRT as predictors [lowest Akaike Information Criteria (AIC) and Bayesian Information Criteria (BIC)] (Supplementary Table 9).

Finally, obesity and hypertension, although not directly associated with increased in-hospital mortality, had a positive correlation with progression of disease severity (i.e., need for MV and/or RRT) (Table 3). Asthma, conversely, was not associated with occurrence of death or progression of disease severity (Table 3).

**Table 3:** Relationship between obesity, asthma, and hypertension with the need for mechanical ventilation and renal replacement therapy in patients with coronavirus 2019 disease from March 1st, 2020 to March 31st, 2021.

Comorbidity	No % (n)	Yes % (n)	Unadjusted OR (CI 95%)	Adjusted OR* (CI 95%)	p-value
**Mechanical ventilation**					
Obesity^a^	86.6 (6995)	13.4 (1086)	1.26 (1.17–1.35)	1.78 (1.56–2.04)	< 0.001
Asthma^a^	89.6 (1555)	10.4 (181)	0.89 (0.76–1.04)	0.99 (0.85–1.18)	0.92
Hypertension^a^	82.5 (11,891)	17.5 (2528)	2.47 (2.32–2.63)	1.09 (1.01–1.17)	0.02
**Renal replacement therapy**					
Obesity^a^	95.5 (7717)	4.5 (364)	1.25 (1.11–1.41)	1.77 (1.64–1.92)	< 0.001
Asthma^a^	97.1 (1686)	2.9 (50)	0.72 (0.54–0.96)	0.84 (0.62–1.15)	0.29
Hypertension^a^	93.3 (13,452)	6.7 (967)	3.46 (3.10–3.86)	1.28 (1.12–1.45)	< 0.001

*Odds ratio adjusted by age group, sex, and other comorbidities through multivariate logistic regression by the enter method.

^a^Values are expressed as relative and absolute frequencies.

## Discussion

To our knowledge, this is the first study to examine the in-hospital mortality and associated risk factors in a large cohort of COVID-19 patients attended exclusively in private Brazilian hospitals. Our observed in-hospital mortality (7.8%) was significantly lower than previously reported rates in Brazil and elsewhere. Of the multiple factors at play, age is likely a major one since increasing age has been consistently associated with higher COVID-19-related mortality. Hence, the lower age (53.2 ± 17.0 years) of our cohort has likely contributed to our observed overall lower in-hospital mortality compared to other large cohort studies (Supplementary Table 10). In comparison, a Middle East study of 23,367 COVID-19 hospitalized patients (age 57.3 ± 17.6 years) reported an overall mortality of 24% (42% in patients > 65 years-old)^12^. In Europe, different mortality rates have been observed depending on the timing of the pandemic and the capacity of healthcare systems. In Germany, for instance, of 10,021 COVID-19 patients (age 72 (IQR 57–82) years) admitted to 920 hospitals, 22% died^13^, whereas a significantly higher in-hospital mortality (39%) was reported among 20,133 patients [age 72.9 (IQR 58–82) years] in the UK during the first wave of the pandemic^14^. In Brazil, three large population-based studies including 522,167 [age 61 (IQR 47–73) years], 254,288 (age 60 ± 17 years) (232,036 of whom had a defined outcome), and 228,196 [age 61 (IQR 48–73) years] COVID-19 patients reported 30.7%^8^, 38%^4^ and 37%^6^ in-hospital mortality, respectively, whereas two smaller cohorts (mean/median ages not reported) of 46,285 and 11,321 in-hospital patients (6882 of whom had a defined outcome) found much higher mortality rates of 46.2%^9^ and 47.3%^7^, respectively (Supplementary Table 10). None of these studies, however, discriminated the in-hospital mortality between public and private hospitals^4,6–10^. In fact, a lower (24.4%) in-hospital mortality was found in a cohort of 89,405 patients (age 58.9 ± 16.8 years) attended exclusively by the Brazilian unified public healthcare system^15^. This study, however, was remarkable for significant underreporting (e.g., nearly 80% of the patients did not have a single comorbidity reported) and contrasting/questionable results (e.g., hypertension and diabetes mellitus (DM) had a protective effect against mortality) when compared to other large population-based studies^15^. Finally, a large (n = 398,063) retrospective cohort of all COVID-19-related hospital admissions between epidemiological weeks 10–40 involving Brazilian public hospitals reported 86,452 (21.7%) deaths with the overall age-standardized in-hospital fatality rate decreasing over time, from 31.8% (95% CI 31.2–32.5%) in week 10 to 18.2% (95% CI 17.6–18.8%) in week 40 of the pandemic^10^. This decreasing trend was observed in all sex, age, ethnic groups, hospital length of stay and ICU admissions^10^. Similarly, the same trend was observed in the private system (Supplementary Table 2), likely reflecting the oscillation of COVID-19 waves, i.e., new case surges followed by case declines.

Apart from age, access to healthcare in Brazil is heterogeneous and may have contributed to our observed lower in-hospital mortality. Important socioeconomic discrepancies have negatively impacted access to an already saturated healthcare system in some regions as indicated by reports on healthcare collapse and disproportional mortality rates^16,17^. Moreover, remarkable disparities between private and public hospitals and the type (and quality) of healthcare available to patients attended by (and dependent on) them are noteworthy. These differences have arguably directly impacted access to intensive care and may explain important imbalances in outcomes observed in critically ill patients^18,19^. In 2020, of the 45,848 adult ICU beds available nationwide, approximately half belonged to the private system, to which less than 25% of the population had access^20,21^. Accordingly, the 35% increase in ICU-bed availability observed at the peak of the pandemic allowed the private health network in question to promptly meet the rapidly increasing demand. In total, 62.5% of the present cohort was admitted to the ICU compared to 33–39% reported in previous large Brazilian population-based studies^4,6,8^. This “elevated” ICU admission rate, however, does not necessarily reflect the overall disease severity of our patients, but rather, a virtually unrestricted access to intensive care which is corroborated by the fact that ICU admission did not correlate with in-hospital mortality (rho = 0.31, *t* = 1.11, p = 0.28) and, in fact, may (or arguably) have contributed to higher survival rates. Similarly, all patients requiring MV were admitted to the ICU and only 0.9% (n = 29) of our observed deaths occurred in a non-intensive/semi-intensive care environment, whereas 14% (n = 5976) of patients requiring MV were ventilated outside the ICU, and 8.9% (n = 7828) of reported deaths occurred in a non-ICU environment in a large (n > 250,000) Brazilian cohort^4^. Finally, 15.4% (n = 15,477) of the COVID-19-related deaths reported in Brazil between Feb and Aug 2020 occurred outside of the hospital environment, which may reflect the oversaturation of the healthcare system resulting in unavailability of hospital/ICU beds^6^.

The lower age (53.2 ± 17.0 years) observed among our patients compared to other large Brazilian cohorts (60 ± 17 years)^4^ likely did not happen at random. Rather, we speculate that younger individuals who are part of the Brazilian workforce are more likely to have access to private health insurance which is often subsidised by employers. Marcolino et al.^22^ recently examined 2054 COVID-19 patients admitted to 25 Brazilian hospitals (12 public, 5 private and 8 “mixed” providing both private and public services) and found a lower median age in the private [55 (IQR 43–67) years] compared to public [59 (IQR 47–71) years] hospitals. Accordingly, the overall in-hospital mortality was significantly lower in private (10.8%) compared to public (24.7%) and “mixed” (26.2%) institutions (p < 0.001)^22^, which corroborates our findings. Similarly, a recent cohort of 13,301 COVID-19 patients [age 54 (IQR 41–69) years] admitted to 126 Brazilian private ICUs showed a 13% mortality^23^, which is somewhat comparable to the 9.1% observed in our cohort. Conversely, substantially higher (51.8^8^–59%^4^) ICU mortality rates have been reported in population-based studies including all Brazilian hospitals (without discriminating between public and private institutions). For reasons already alluded to (e.g., chronic underfunding of public hospitals, better infrastructure and more qualified personnel on the private sector, etc.), it is reasonable to assume that such a large gap would become even more pronounced in an analysis (currently not available) focused at distinctively examining the in-hospital mortality between public and private Brazilian institutions. Indeed, when compared to > 250,000 COVID-19 patients admitted to (all) Brazilian hospitals between Feb-Aug 2020, the proportion of deaths in our cohort was substantially lower in all age groups, being more pronounced in patients between 20 and 39 years-old (88% mortality reduction), and less so (though still significant) in patients ≥ 80 years-old (25% reduction) (p < 0.001) (Supplementary Table 11).

In the present cohort, MV was instituted in 4467 (11.5%) patients, of whom 59.6% died (HR 11.89, p < 0.001) (Supplementary Fig. 7), which highlights the importance of MV as a predictor of death in patients hospitalized due to COVID-19. Mortality rates in mechanically ventilated patients due to COVID-19 have been reported at 52.8% in Europe^13^, 74% in Mexico^24^, and staggering 80% in Brazil^4^ (Supplementary Table 10). Such large variability is likely multifactorial, including local infrastructure, availability (and qualification) of human (as well as equipment, pharmacologic and technological) resources, combined with patients’ underlying socioeconomic and health (i.e., comorbidities) conditions^24^. Notably, in countries like Brazil with drastic socioeconomic disparities, access to private healthcare arguably allows for better (and ongoing) assistance which may (and likely do) contribute to better optimization of chronic conditions/comorbidities, thereby potentially impacting COVID-19-related survival rates.

Although the pathophysiology of COVID-19 remains not completely elucidated, it has been well documented that older patients and those with comorbidities present higher mortality rates^25,26^, which was corroborated by our findings (Table 1, Fig. 2, Supplementary Fig. 8, and Supplementary Table 6). Indeed, a British study showed that heart disease, chronic lung disease, obesity, DM, and immunosuppression presented statistically significant hazard ratios in relation to the time of progression to death^14^.

Obesity has been consistently associated with an increased disease burden in COVID-19 patients. The relationship between obesity and death, however, remains unclear, as available data have been accrued from heterogeneous studies with different population characteristics and variable study designs^10,27,28^. A large cohort study examined the risk factors associated with in-hospital mortality in 17 million adult patients with COVID-19 and showed that even though grade I obesity was not associated with the occurrence of death, the risk progressively increased in more obese patients, with the highest risk in those with morbid obesity^27^. Conversely, a Mexican study showed no relationship between obesity and in-hospital mortality in COVID-19 patients^24^. In the present investigation, a positive correlation was observed between obesity and the need for MV and/or RRT, which in turn, have been associated with poor prognosis in COVID-19 patients, even after adjustment for age and other comorbidities^29^.

In this study, hypertension was not associated with increased risk of death in COVID-19 patients, which corroborates previous findings^27,30^. The definitive link between hypertension and mortality in COVID-19, however, remains unclear^31^. All in all, the precise effect of each comorbidity on the final outcome of COVID-19 is virtually impossible to determine, and it is likely that the duration of a certain comorbidity and, perhaps most importantly, whether adequate (long-term) disease control has been attained should be included in this complex equation of potential risk factors associated with poor outcomes and mortality in COVID-19.

This study has several limitations. First, given the dynamic nature of the pandemic, clinical practices were changed and improvements were implemented over time as a better understanding of the disease was progressively unveiled during the pandemic. Evaluation of such temporal changes (which may have affected in-hospital mortality) was beyond the scope of this study. Secondly, since this was a retrospective database study, the final analyses were not adjusted to some patient characteristics, such as race, income, level of education, nutritional status, length of illness prior to hospitalization, severity scores of clinical symptoms prior to and during hospitalization, in-hospital investigations and implemented management measures. Thirdly, the (non-standardized) indications for non-invasive ventilatory support and use of supplemental oxygen were not included in this analysis and may have influenced the institution of MV across participating institutions^13^. Fourthly, the effect of SARS-CoV-2 virus variants was not accounted for in our results. Finally, our results may not be generalizable as this study only included patients who had access to 52 private hospitals in Brazil (which are not necessarily representative of the entire Brazilian private healthcare system) where the supply of resources met the rapidly increasing demand throughout the studied period, which does not reflect the reality of the great majority of the Brazilian population who rely (and depend exclusively) on the public healthcare system. Nevertheless, years (or even decades) worth of life expectancy gains in Brazil have been rapidly reversed by the pandemic^32^, and the significant difference in in-hospital mortality between Brazilian hospitals in general (38%)^4^ and the local private healthcare (7.8%), although multifactorial, arguably reflects the historical socioeconomic abyss existent in the country. Of many factors at play, more investments in infrastructure and healthcare professionals’ qualification (as well as ensuring an adequate workload) is key, especially in the public system, to start closing the gap.

In summary, the observed in-hospital mortality due to COVID-19 was 7.8% in this large cohort (N = 38,937) of patients admitted exclusively to Brazilian private hospitals. Age ≥ 61 years-old, the presence and number of comorbidities (i.e., cardiovascular disease, chronic kidney disease (CKD), chronic obstructive pulmonlary disease (COPD), DM, cerebrovascular disease, and immunosuppression), and the need for MV and/or RRT were independently associated with in-hospital mortality during the first 13 months of the pandemic in Brazil.

## Methods

**Figure 3:**
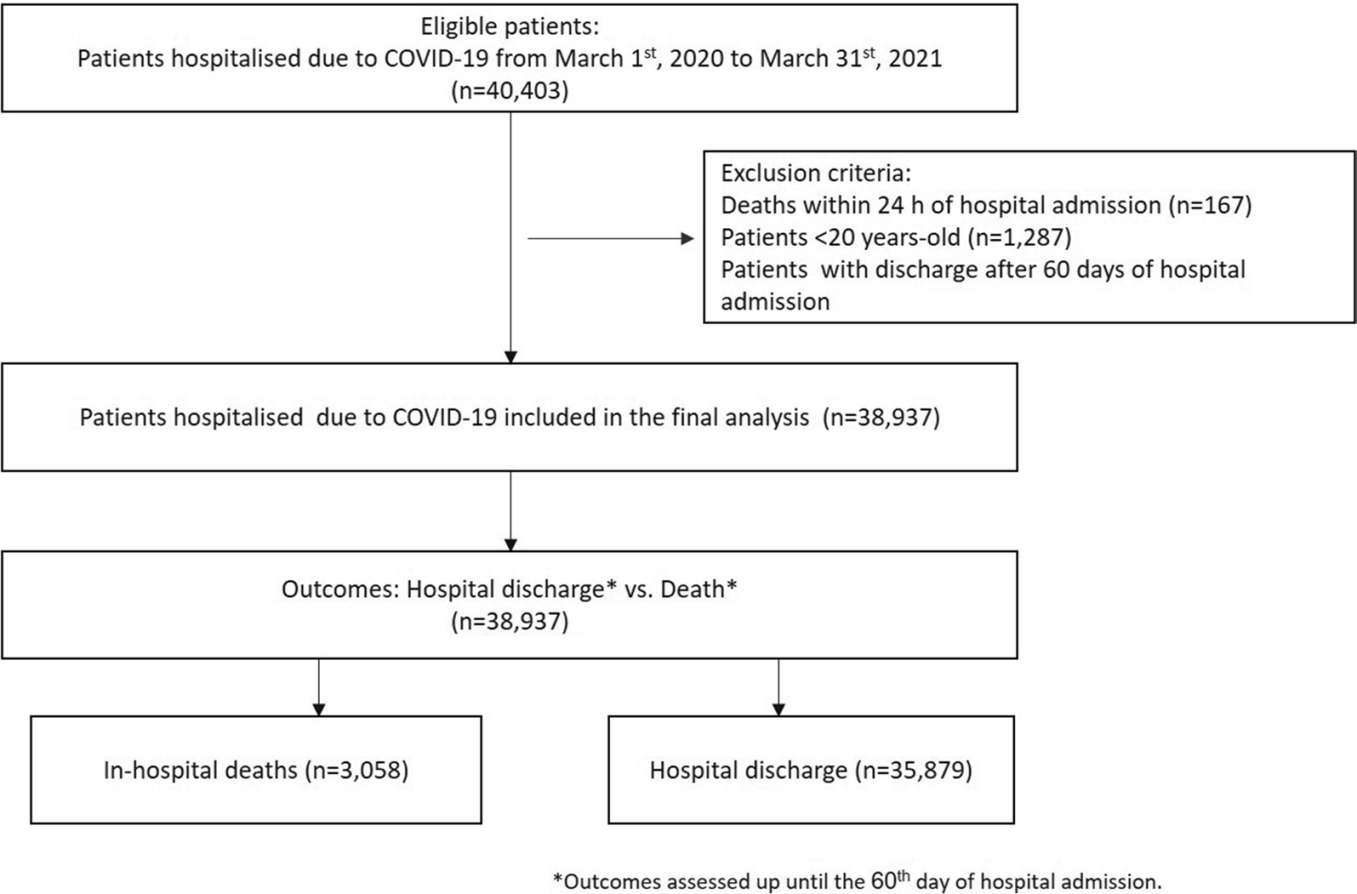
Diagram showing patient exclusions and the distribution of patients included in the final analysis according to outcomes (i.e. in-hospital death vs. hospital discharge).

This was a longitudinal retrospective cohort study of patients ≥ 20 years-old who were hospitalized due to COVID-19 in 52 hospitals comprising a private Brazilian healthcare network (Rede D’Or São Luiz). Participating institutions were tertiary hospitals distributed across 4 (Northeast, Central-West, Southeast, and South) of Brazil’s 5 macro geographical regions. Data were extracted from the institutional central database comprised of COVID-19 diagnoses and related deaths (both of which are of compulsory notification to the BMH). Participating hospitals utilized a standardized data collection model developed at the early stages of the pandemic by the Quality Board of the Rede D’Or network and implemented in all of its units/hospitals with the aims to (1) standardize data collection pertaining to COVID-19; (2) optimize resource allocation, given that the pandemic was not equally distributed across the vast Brazilian territory; and (3) adequately report data of compulsory notification (e.g., COVID-19 diagnosis and related deaths) to the BMH. All nurses who collected COVID-19-related data/information from each institution were specifically trained for this purpose. Data from each participating institution were continuously collected and, upon compilation, submitted weekly to a central database where all data pertaining to the present investigation were extracted from. The study period included the first 13 months of the pandemic, i.e., from March 1st, 2020 to March 31st, 2021. All patients hospitalized due to COVID-19 (as the primary diagnosis upon admission) who were either discharged or died were included (Fig. 3).

The primary outcome consisted of hospital LOS (days) until either death or hospital discharge, and was measured up until the 60th day of hospital admission. A positive result for the reverse transcription polymerase chain reaction (RT-PCR) diagnostic test for the SARS-CoV-2 virus was defined as a confirmed case.

To identify risk factors associated with the occurrence of death, the survival time was modeled based on the following independent variables: age (years), sex, comorbidities [i.e., DM, cardiovascular disease, cerebrovascular disease, hypertension, obesity, COPD, asthma, CKD, and immunosuppression], and the need for MV and/or RRT. Demographic data were obtained upon hospital admission. Definitions included: cardiovascular disease: cardiomyopathy and/or heart failure of any etiology (e.g., ischemic, arrhythmias); cerebrovascular disease: previous stroke or transient ischemic attack; immunosuppression: cancer diagnosis within the previous 6 months, ongoing oncologic treatment, or diseases/conditions (e.g., systemic steroid use) affecting the immune response. Additionally, self-reported chronic diseases requiring pharmacologic treatment (e.g., hypercholesterolemia) were recorded as comorbidities.

This investigation was approved by the local Institutional Research Ethics Board (IREB) (protocol #45576521.8.0000.0087, assent CEP4.659.131) and all methods were performed in accordance with the relevant guidelines and regulations. Specifically, the STROBE guidelines were followed (Supplementary Material) and this study complied with the Resolution 466/2012 of the Brazilian National Health Council. In addition, informed consent was waived by the Hospital São Luiz & Rede D’or and Affiliated Teaching Hospitals Research Ethics Board.

### Statistical analysis

Statistical analysis was performed using the Stata/SE 16·0 for Windows software (Stata Corporation, College Station, Texas, USA). Categorical variables are presented as absolute values and percentages, and continuous variables are represented as means and standard deviations or medians and 25–75% percentiles, as appropriate. Histograms and the Shapiro–Wilk test were used to assess data distribution. Spearman’s test was employed for correlation analysis between in-hospital deaths and the need for MV or ICU admission. The survival analysis considered the occurrence of death in relation to the duration of hospitalization as the dependent variable. Survival curves were generated by the Kaplan–Meier method, and the Log-rank test was used to compare groups for each variable. Cox proportional-hazards model was used to estimate the risk of death by study variables. The assumption of proportionality of variables was verified by Kaplan–Meier curves and Schoenfeld residual analysis. The multivariate model included variables that presented a significance level of p < 0·20 in the univariate analysis. The stepwise backward method was used from the saturated model, until the model with the fewest variables that would explain most of the variance was identified. Model adjustment was performed by the likelihood ratio of the proposed model relative to the saturated model. Associations between study variables and death are expressed as hazard ratios (HR) with 95% confidence intervals (CI). The final multivariate model accepted a significance level of p < 0.05. The comparison between predictive models was assessed using HR, AIC and BIC, with the lowest AIC and BIC indicative of the best fit. The analysis of comorbidities not related to death during hospitalization is presented as odds ratios (OR) with 95% CI as a measure of the association between each comorbidity and the indicators adopted for progression of disease severity, namely (i) need for MV and/or (ii) RRT (both dichotomous dependent variables), with adjustments for age, sex and other comorbidities. Multivariate logistic regression by the direct method was used for this purpose.

### Ethics

Ethical approval was obtained from the Hospital São Luiz & Rede D’or and Affiliated Teaching Hospitals Research Ethics Board for publication of this report (protocol #45576521.8.0000.0087, assent CEP4.659.131). Additionally, this study complied with the Resolution 466/2012 of the Brazilian National Health Council.

## Supplementary Information

Supplementary Information.

## Data Availability

The datasets generated during and/or analyzed during the current study are available in Mendeley Data [Muniz da Silva, Leopoldo (2022), “DATABASECOVID2021”, Mendeley Data, V1, https://data.mendeley.com/datasets/7xsvwsxbtd/1]. The files associated with this dataset are licensed under an attribution non-commercial 3.0 Unported license (CC BY NC 3.0).
