# Supplementary material for: Coronavirus disease-related in-hospital mortality: a cohort study in a private healthcare network in Brazil
Source: Sci Rep. 2022 Apr 16;12:6371. doi: 10.1038/s41598-022-10343-4 (PMC9012947; doi:10.1038/s41598-022-10343-4)
Supplement: Supplementary file 1 — Supplementary Information. [file 41598_2022_10343_MOESM1_ESM.docx]

**CORONAVIRUS DISEASE-RELATED IN-HOSPITAL MORTALITY: A cohort study in a private healthcare network in Brazil**

**Electronic Supplementary Material**

Lima et al.

| **Table of contents** | |  |
| --- | --- | --- |
| **Supplementary Files** | **Figure &Table Headings** | **Page** |
| **Supplementary Figure 1** | **Geographical distribution of patients hospitalized due to COVID-19 in Brazil from March 1^st^, 2020 to March 31^st^, 2021. State acronyms by region: Northeast: BA = Bahia, CE = Ceará, MA = Maranhão, PE = Pernambuco, SE = Sergipe; Central-West: DF = Distrito Federal; Southeast: RJ = Rio de Janeiro, SP = São Paulo; South: PR = Paraná. This map was generated using Microsoft Excel 2019 MSO (version 2201 Build 16.0.14827.20198) 64 bits, powered by Bing. @DSAT for MSFT, Geonames, Microsoft, Navteq (**[**https://www.microsoft.com/en-us/maps/product/print-rights**](https://www.microsoft.com/en-us/maps/product/print-rights)**).** | **4** |
| **Supplementary Figure 2** | **Patient distribution according to age groups (years) and the studied outcomes, i.e., hospital discharge (left panel) and in-hospital death (right panel) from March 1^st^, 2020 (Month 1) to March 31^st^, 2021 (Month 13). The lines in the box represent the mean (central line) and standard deviation (upper and lower lines).** | **4** |
| **Supplementary Figure 3** | **Correlation between in-hospital deaths and intensive care unit (ICU) admissions, need for mechanical ventilation, hospital admissions and ICU-bed availability in patients hospitalized due to COVID-19 from March 1^st^, 2020 to March 31^st^, 2021 (Spearman correlation).** | **5** |
| **Supplementary Figure 4** | **In-hospital mortality stratified by hospital allocation (ICU, Step down unit, Ward) and age (years) in patients hospitalized due to COVID-19 from March 1^st^, 2020 to March 31^st^, 2021.** **The lines in the box represent the mean (central line) and standard deviation (upper and lower lines). ANOVA with Bonferroni Correction: p=0.001; ICU (age 79.3±14.4) > Step down unit (age 69.4±15.4) and Ward (age 72.4±15.6). ICU = intensive care unit.** | **5** |
| **Supplementary Figure 5** | **Comorbidities of patients hospitalized due to COVID-19 requiring extracorporeal membrane oxygenation (n=79) from March 1^st^, 2020 to March 31^st^, 2021. Values expressed as %.** | **6** |
| **Supplementary Figure 6** | **Distribution of patients hospitalized due to COVID-19 requiring extracorporeal membrane oxygenation (n=79) from March 1^st^, 2020 to March 31^st^, 2021 according to age groups (years), duration of hospitalization (days) and studied outcomes (i.e., hospital discharge and in-hospital death). The lines in the box represent the mean (central line) and standard deviation (upper and lower lines). Unpaired two-sample t-tests.** | **6** |
| **Supplementary Figure 7** | **Kaplan-Meier curves showing the probability (%) of survival over the first 60 days of hospitalization due to coronavirus 2019 disease from March 1^st^, 2020 to March 31^st^, 2021 in relation to comorbidities that reached statistical significance in the final model. Log-rank test: p<0·05 for each variable.** | **7** |
| **Supplementary Figure 8** | **Kaplan-Meier curves showing the probability (%) of survival over the first 60 days of hospitalization due to coronavirus 2019 disease from March 1^st^, 2020 to March 31^st^, 2021 in relation to age groups, number of comorbidities, and need for mechanical ventilation and renal replacement therapy. Log-rank test: p<0·05 for each variable.** | **7** |
| **Supplementary Table 1** | **Hospital discharge in patients hospitalized due to COVID-19 from March 1^st^, 2020 to March 31^st^, 2021** **(Proportion – 95%CI).** | **8** |
| **Supplementary Table 2** | **In-hospital deaths in patients hospitalized due to COVID-19 from March 1^st^, 2020 to March 31^st^, 2021 (Proportion – 95%CI).** | **8** |
| **Supplementary Table 3** | **Demographic variables of patients hospitalized due to COVID-19 requiring extracorporeal membrane oxygenation from March 1^st^, 2020 to March 31^st^, 2021. Unadjusted analysis using the multivariate logistic regression model.** | **9** |
| **Supplementary Table 4** | **Distribution of hospital discharges and in-hospital deaths according to the presence and number of comorbidities in patients hospitalized due to COVID-19 requiring extracorporeal membrane oxygenation from March 1^st^, 2020 to March 31^st^, 2021. Unadjusted analysis using logistic regression model.** | **9** |
| **Supplementary Table 5** | **Distribution of hospital discharges and in-hospital deaths according to the presence and number of comorbidities in patients hospitalized due to COVID-19 requiring extracorporeal membrane oxygenation from March 1^st^, 2020 to March 31^st^, 2021. Adjusted analysis using logistic regression model (Stepwise method).** | **10** |
| **Supplementary Table 6** | **In-hospital mortality stratified by age range and number of comorbidities in patients hospitalized due to COVID-19 from March 1^st^, 2020 to March 31^st^, 2021.** | **10** |
| **Supplementary Table 7** | **In-hospital mortality stratified by age range and the need for mechanical ventilation in patients hospitalized due to COVID-19 from March 1^st^, 2020 to March 31^st^, 2021.** | **11** |
| **Supplementary Table 8** | **In-hospital mortality stratified by age range and the need for renal replacement therapy in patients hospitalized due to COVID-19 from March 1^st^, 2020 to March 31^st^, 2021.** | **11** |
| **Supplementary Table 9** | **Comparison of four multivariable logistic regression modelling approaches in combination with goodness of-fit measures (Akaike information criteria and Bayesian Information Criteria).** | **11** |
| **Supplementary Table 10** | **Comparison of large COVID-19 cohorts.** | **12–13** |
| **Supplementary Table 11** | **Proportion of deaths stratified by age range: Comparison of two large Brazilian cohorts (Ranzani^1^ et al vs. Lima et al) of patients hospitalized due to COVID-19.** | **14** |
| **References** | **N/A** | **15** |
| **STROBE checklist** | **N/A** | **16-17** |

# **Supplementary Contents**


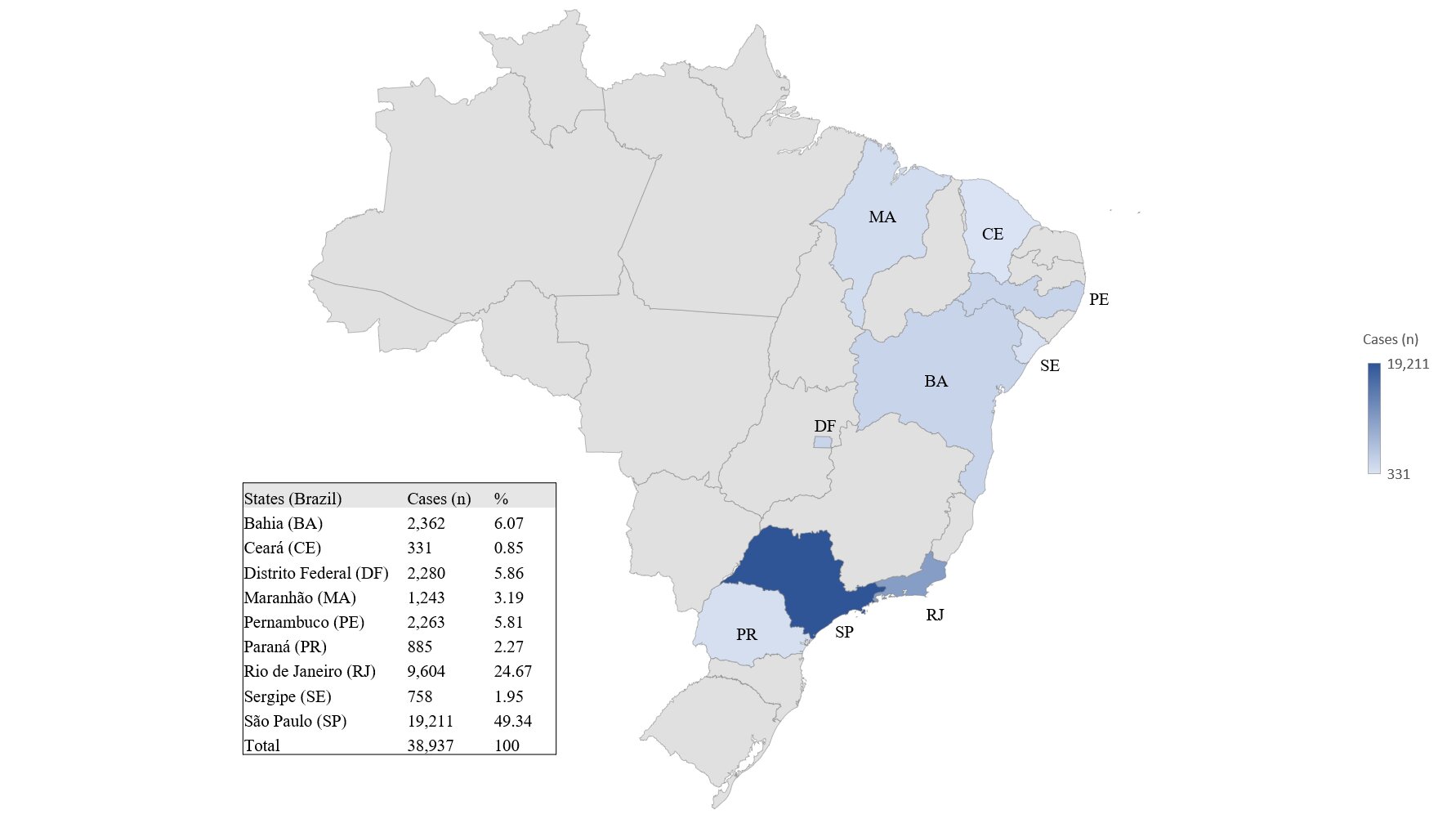


**Supplementary Figure 1. Geographical distribution of patients hospitalized due to COVID-19 in Brazil from March 1^st^, 2020 to March 31^st^, 2021. State acronyms by region: Northeast: BA = Bahia, CE = Ceará, MA = Maranhão, PE = Pernambuco, SE = Sergipe; Central-West: DF = Distrito Federal; Southeast: RJ = Rio de Janeiro, SP = São Paulo; South: PR = Paraná. This map was generated using Microsoft Excel 2019 MSO (version 2201 Build 16.0.14827.20198) 64 bits, powered by Bing. @DSAT for MSFT, Geonames, Microsoft, Navteq (**[**https://www.microsoft.com/en-us/maps/product/print-rights**](https://www.microsoft.com/en-us/maps/product/print-rights)**).**


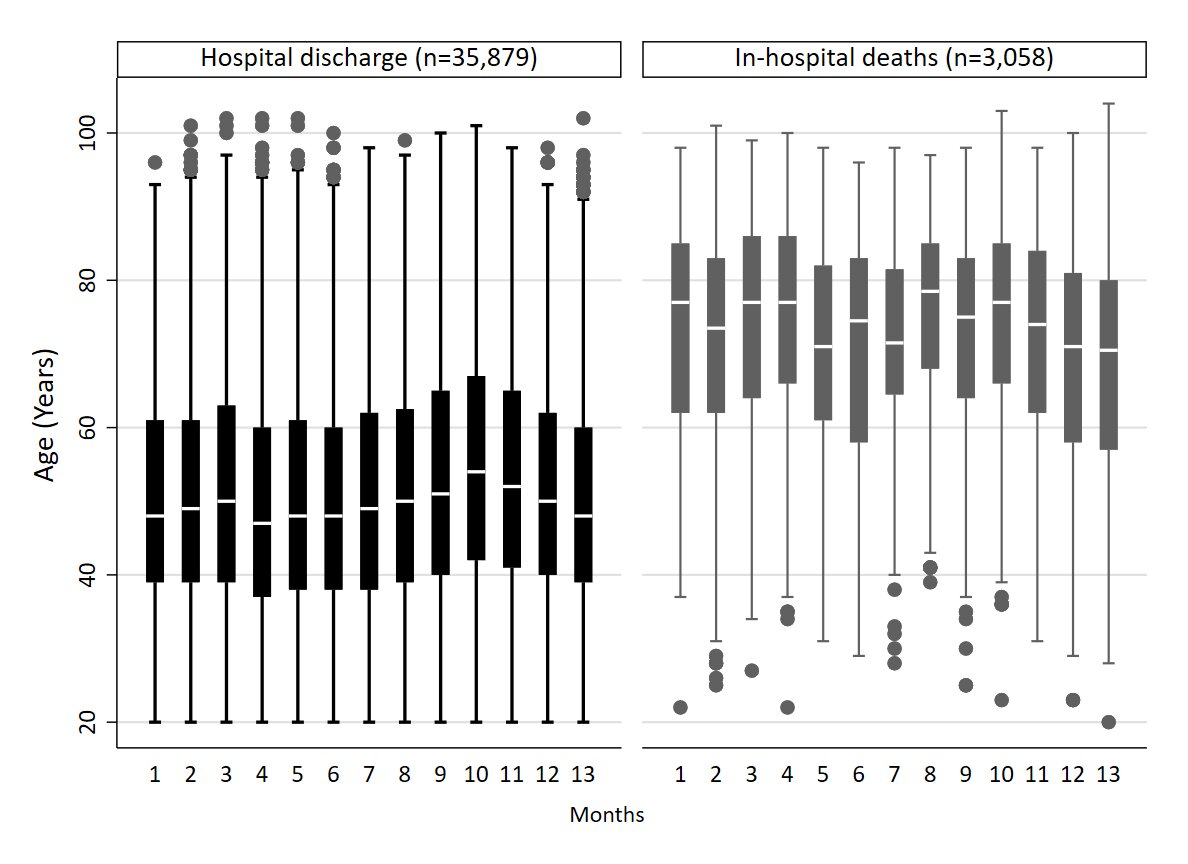


**Supplementary Figure 2. Patient distribution according to age groups (years) and the studied outcomes, i.e., hospital discharge (left panel) and in-hospital death (right panel) from March 1^st^, 2020 (Month 1) to March 31^st^, 2021 (Month 13). The lines in the box represent the mean (central line) and standard deviation (upper and lower lines).**


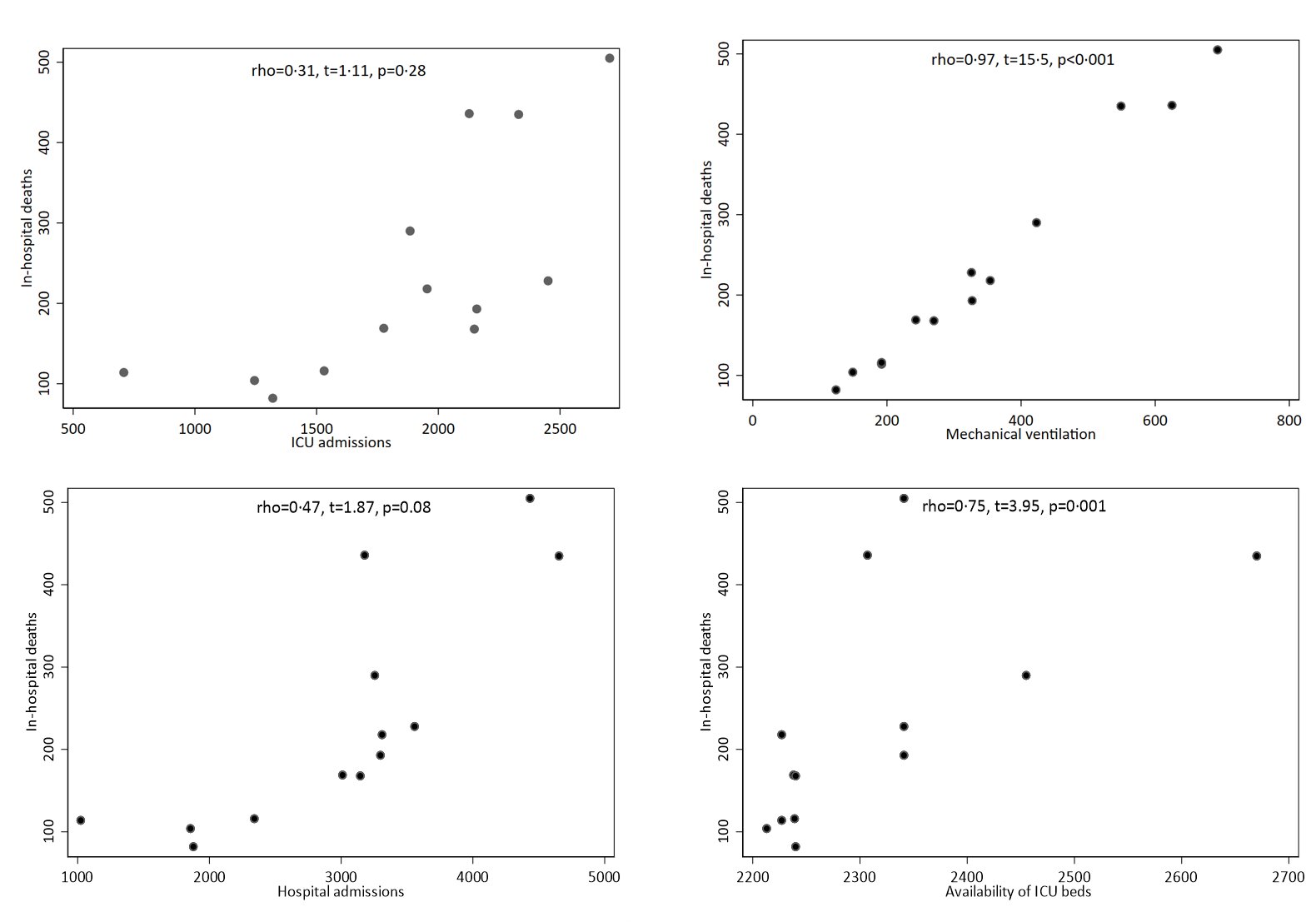


**Supplementary Figure 3.** **Correlation between in-hospital deaths and intensive care unit (ICU) admissions, need for mechanical ventilation, hospital admissions and of ICU-bed availability in patients hospitalized due to COVID-19 from March 1^st^, 2020 to March 31^st^, 2021 (Spearman correlation).**

**Supplementary Figure 4. In-hospital mortality stratified by hospital allocation (ICU, Step down unit, Ward) and age (years) in patients hospitalized due to COVID-19 from March 1^st^, 2020 to March 31^st^, 2021.** **The lines in the box represent the mean (central line) and standard deviation (upper and lower lines). ANOVA with Bonferroni Correction: p=0.001; ICU (age 79.3±14.4) > Step down (age 69.4±15.4) and Ward (age 72.4±15.6). ICU = intensive care unit.**


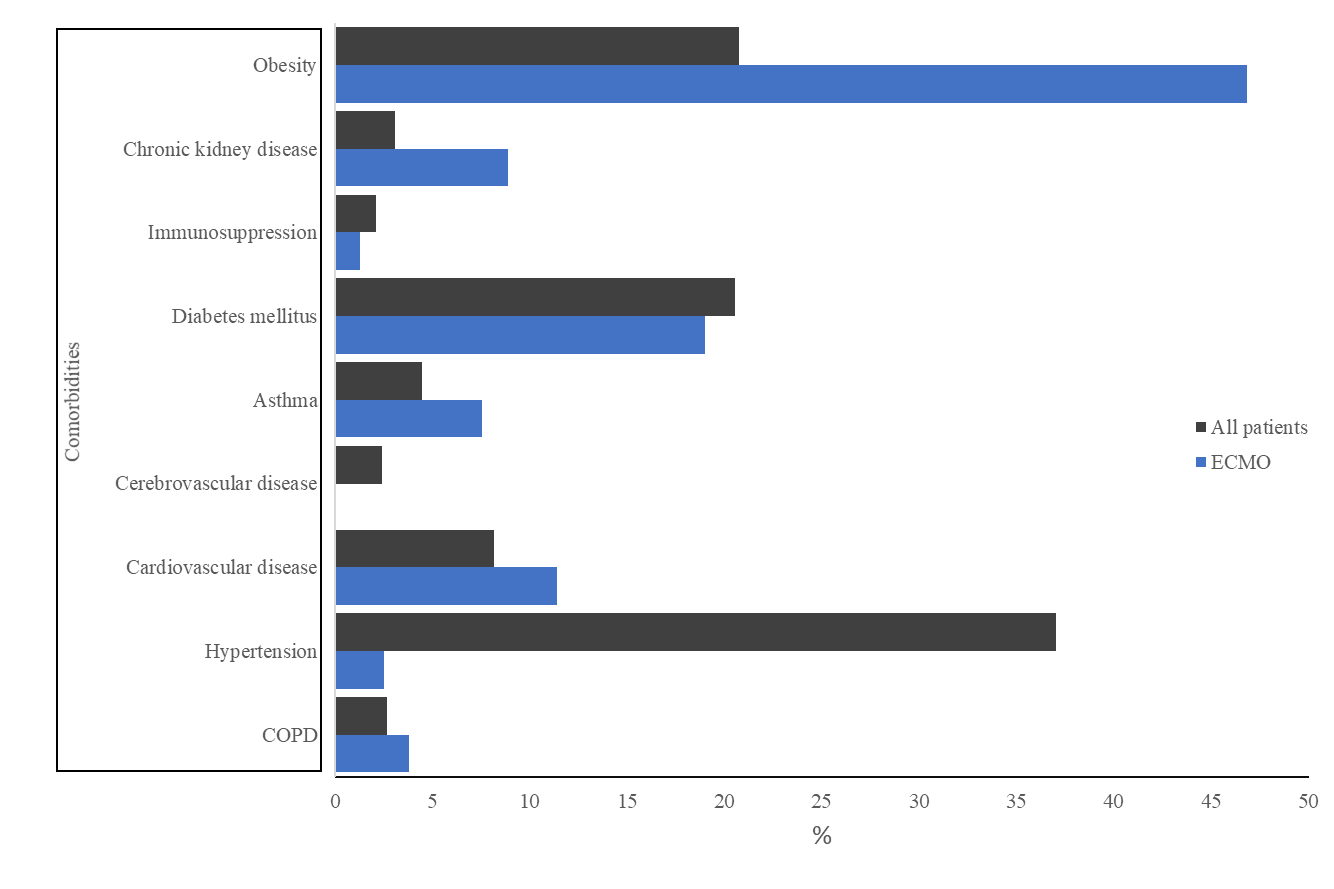


**Supplementary Figure 5.** **Comorbidities of patients hospitalized due to COVID-19 requiring extracorporeal membrane oxygenation (n=79) from March 1^st^, 2020 to March 31^st^, 2021. Values expressed as %.**


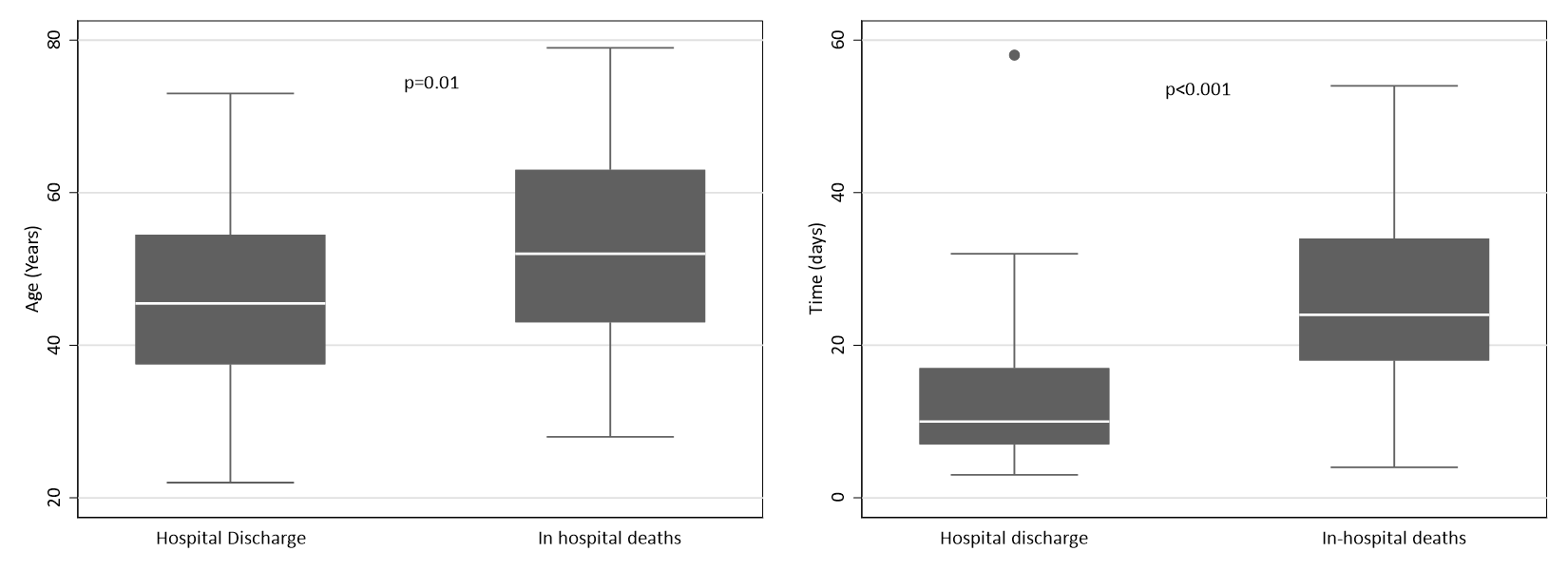


**Supplementary Figure 6. Distribution of patients hospitalized due to COVID-19 requiring extracorporeal membrane oxygenation (n=79) from March 1^st^, 2020 to March 31^st^, 2021 according to age groups (years), duration of hospitalization (days) and studied outcomes (i.e., hospital discharge and in-hospital death). The lines in the box represent the mean (central line) and standard deviation (upper and lower lines). Unpaired two-sample t-tests.**


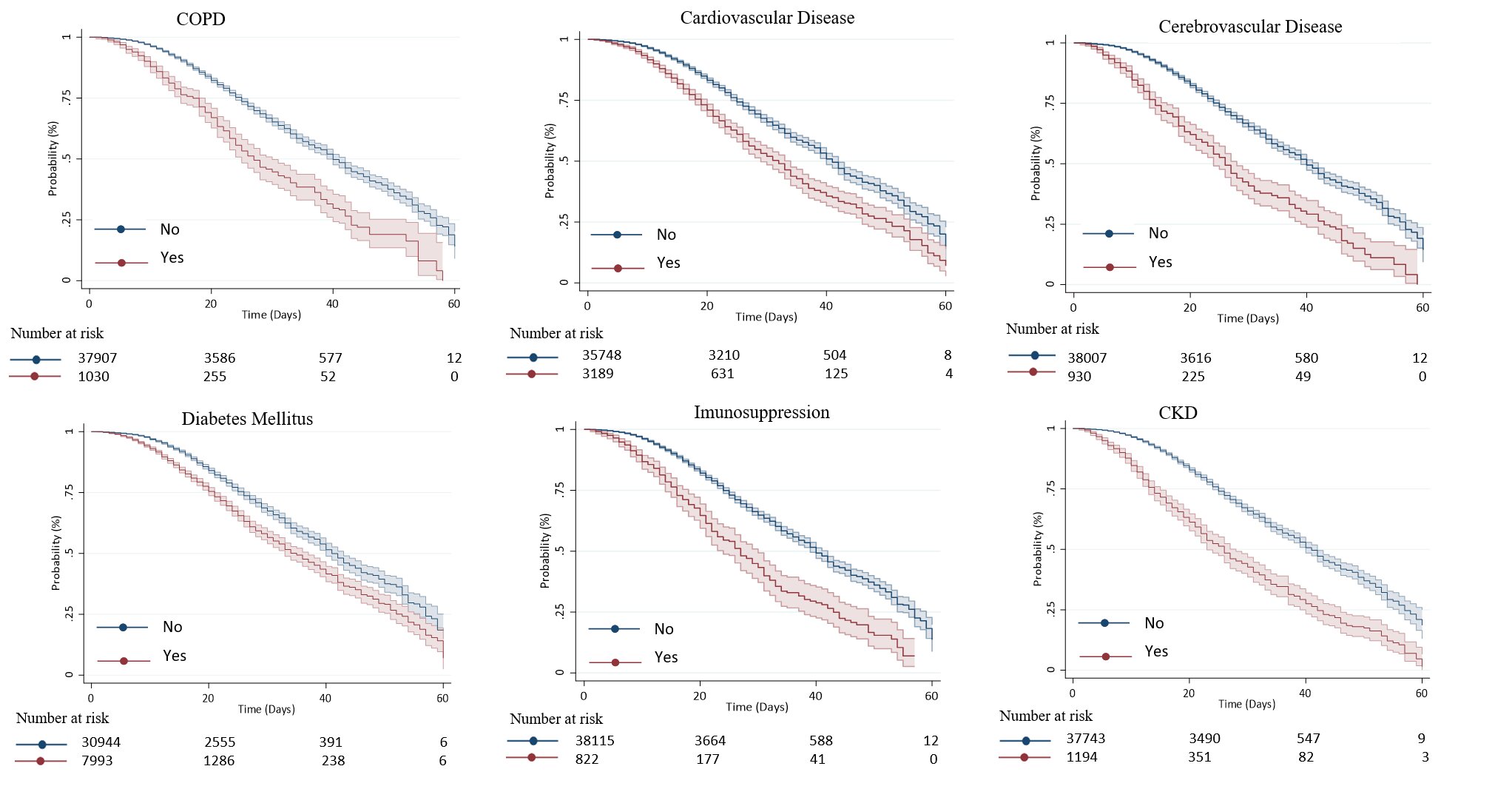


**Supplementary Figure 7. Kaplan-Meier curves showing the probability (%) of survival over the first 60 days of hospitalization due to coronavirus 2019 disease from March 1^st^, 2020 to March 31^st^, 2021 in relation to comorbidities that reached statistical significance in the final model. Log-rank test: p<0·05 for each variable.**


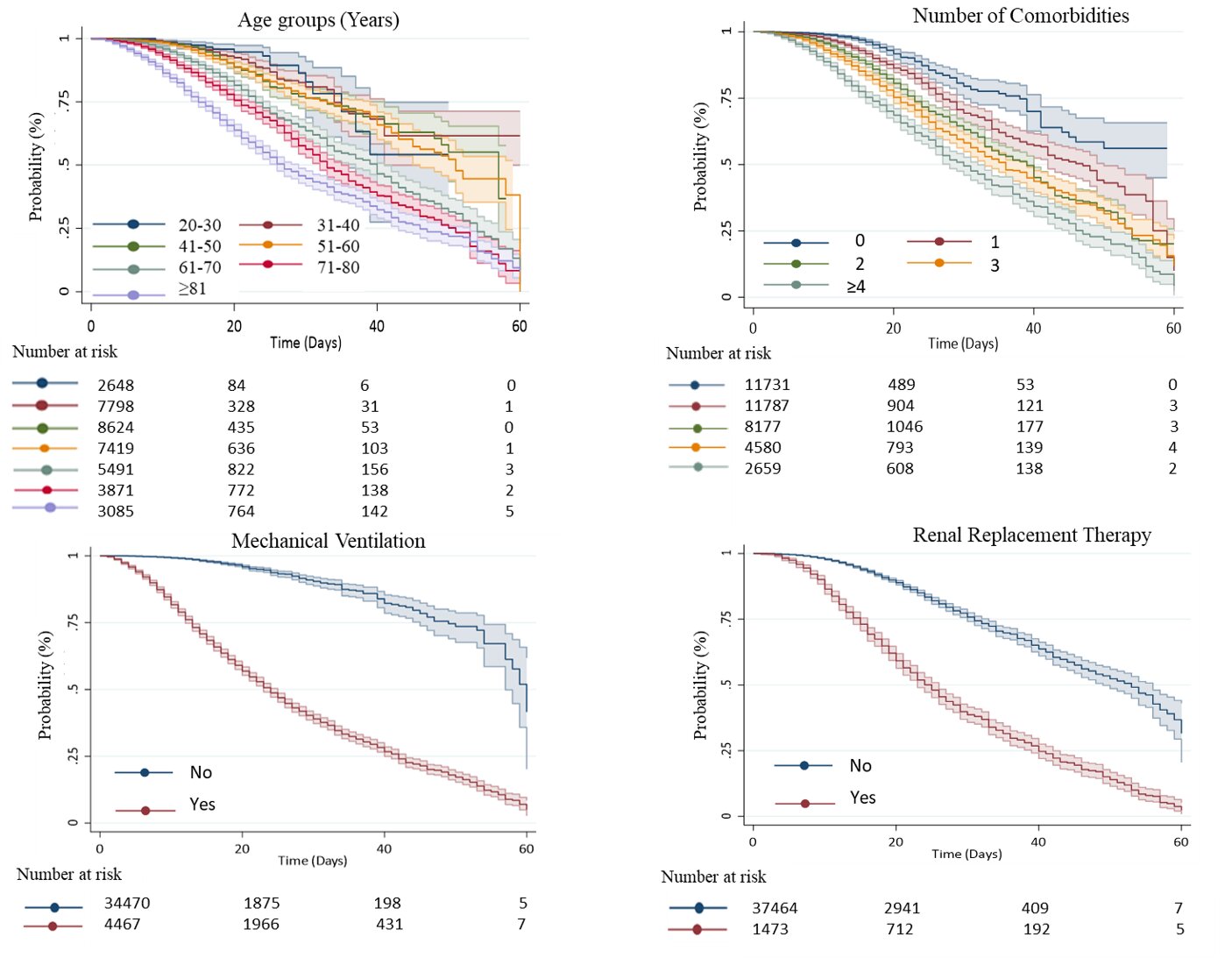


**Supplementary Figure 8. Kaplan-Meier curves showing the probability (%) of survival over the first 60 days of hospitalization due to coronavirus 2019 disease from March 1^st^, 2020 to March 31^st^, 2021 in relation to age groups, number of comorbidities, and need for mechanical ventilation and renal replacement therapy. Log-rank test: p<0·05 for each variable.**

**Supplementary Table 1.** **Hospital discharge in patients hospitalized due to COVID-19 from March 1^st^, 2020 to March 31^st^, 2021 (Proportion – 95%CI).**

| **Month-Year** | **Hospital discharge (n)** | **Proportion** | **95%CI Lower limit** | **95%CI Upper limit** |
| --- | --- | --- | --- | --- |
| March 2020 | 890 | 0.8865 | 0.8653 | 0.9047 |
| April 2020 | 2,749 | 0.8631 | 0.8507 | 0.8746 |
| May 2020 | 3,926 | 0.8860 | 0.8763 | 0.8951 |
| June 2020 | 3,075 | 0.9338 | 0.9248 | 0.9418 |
| July 2020 | 2,858 | 0.9442 | 0.9354 | 0.9518 |
| August 2020 | 2,223 | 0.9504 | 0.9408 | 0.9585 |
| September 2020 | 1,715 | 0.9428 | 0.9312 | 0.9526 |
| October 2020 | 1,756 | 0.9554 | 0.9449 | 0.9640 |
| November 2020 | 2,990 | 0.9468 | 0.9384 | 0.9541 |
| December 2020 | 3,397 | 0.9371 | 0.9287 | 0.9446 |
| January 2021 | 3,117 | 0.9417 | 0.9332 | 0.9492 |
| February | 2,918 | 0.9096 | 0.8992 | 0.9191 |
| March 2021 | 4,265 | 0.9074 | 0.8988 | 0.9154 |
| Total | 35,879 | 0.9215 | 0.9187 | 0.9241 |

**Upper and lower 95%CI limits for in-hospital discharge, estimated by the Agresti-Coull method. CI = Confidence interval.**

**Supplementary Table 2. In-hospital deaths in patients hospitalized due to COVID-19 from March 1^st^, 2020 to March 31^st^, 2021 (Proportion – 95%CI).**

| **Month-Year** | **In-hospital deaths (n)** | **Proportion** | **95%CI Lower limit** | **95%CI Upper limit** |
| --- | --- | --- | --- | --- |
| March 2020 | 114 | 0.1135 | 0.0953 | 0.1347 |
| April 2020 | 436 | 0.1369 | 0.1254 | 0.1493 |
| May 2020 | 505 | 0.1140 | 0.1049 | 0.1237 |
| June 2020 | 218 | 0.0662 | 0.0582 | 0.0752 |
| July 2020 | 169 | 0.0558 | 0.0482 | 0.0646 |
| August 2020 | 116 | 0.0496 | 0.0415 | 0.0592 |
| September 2020 | 104 | 0.0572 | 0.0474 | 0.0688 |
| October 2020 | 82 | 0.0446 | 0.0360 | 0.0551 |
| November 2020 | 168 | 0.0532 | 0.0459 | 0.0616 |
| December 2020 | 228 | 0.0629 | 0.0554 | 0.0713 |
| January 2021 | 193 | 0.0583 | 0.0508 | 0.0668 |
| February | 290 | 0.0904 | 0.0809 | 0.1008 |
| March 2021 | 435 | 0.0926 | 0.0846 | 0.1012 |
| Total | 3,058 | 0.0785 | 0.0759 | 0.0813 |

**Upper and lower 95%CI limits for in-hospital mortality, estimated by the Agresti-Coull method. CI = Confidence interval.**

**Supplementary Table 3. Demographic variables of patients hospitalized due to COVID-19 requiring extracorporeal membrane oxygenation from March 1^st^, 2020 to March 31^st^, 2021. Unadjusted analysis using the multivariate logistic regression model.**

| **Characteristics** | **Unadjusted univariate analysis** | | | | |
| --- | --- | --- | --- | --- | --- |
|  | **Discharge % (n)** | **Death % (n)** | **OR** | **95%CI** | **p-value** |
| Total | 45.6 (36) | 54.4 (43) | - | - | - |
| Age groups (Years)^1^ |  |  |  |  |  |
| 20-30 | 13.9 (5) | 2.3 (1) | 1.00 | - | - |
| 31-40 | 25.0 (9) | 18.6 (8) | 4.44 | 0.42-46.5 | 0.21 |
| 41-50 | 33.3 (12) | 23.3 (10) | 4.16 | 0.41-41.7 | 0.22 |
| 51-60 | 5.6 (2) | 1.·3 (7) | 17.49 | 1.22-25.0 | 0.03 |
| 61-70 | 13.9 (5) | 30.2 (13) | 12.99 | 1.2-14.0 | 0.03 |
| 71-80 | 8.3 (3) | 9.3 (4) | 6.66 | 0.48-9.1 | 0.15 |
| ≥81 | 0.0 (0) | 0.0 (0) | N/A | N/A | N/A |
| Sex^1^ |  |  |  |  |  |
| Female | 38.9 (14) | 34.9 (15) | 1.00 | - | - |
| Male | 61.1 (22) | 65.1 (28) | 1.07 | 0.47-2.97 | 0.19 |
| Geographic distribution^1^ |  |  |  |  |  |
| Central-West | 11.1 (4) | 23.2 (10) | 1.00 |  |  |
| Northeast | 2.8 (1) | 7.0 (3) | 0.83 | 0.06-10.59 | 0.88 |
| Southeast | 86.1 (31) | 69.8 (30) | 0.32 | 0.03-3.27 | 0.33 |
| South | 0.0 (0) | 0.0 (0) | N/A | N/A | N/A |

**^1^Values expressed as relative and absolute frequencies. OR = Odds ratio. CI = Confidence interval.**

**Supplementary Table 4. Distribution of hospital discharges and in-hospital deaths according to the presence and number of comorbidities in patients hospitalized due to COVID-19 requiring extracorporeal membrane oxygenation from March 1^st^, 2020 to March 31^st^, 2021. Unadjusted analysis using logistic regression model.**

| **Comorbidity*** | **Unadjusted univariate analysis** | | | | |
| --- | --- | --- | --- | --- | --- |
|  | **Discharge** | **Death** | **OR** | **95%CI** | **p-value** |
| Chronic obstructive pulmonary disease ^1^ | 0.0 (0) | 7.0 (3) | N/A |  |  |
| Asthma^1^ | 5.6 (2) | 9.3 (4) | 1.74 | 0.30-10.12 | 0.62 |
| Cardiovascular disease^1^ | 5.6 (2) | 16.3 (2) | 3.30 | 0.64-17.6 | 0.15 |
| Cerebrovascular disease^1^ | 0.0 (0) | 0.0 (0) | N/A |  |  |
| Hypertension^1^ | 33.3 (12) | 48.8 (21) | 1.90 | 0.76-4.76 | 0.16 |
| Diabetes mellitus^1^ | 8.3 (3) | 27.9 (12) | 4.25 | 1.09-6.53 | 0.03 |
| Obesity (BMI>30kg/m^2^)^1^ | 47.2 (17) | 46.5 (20) | 0.97 | 0.40-2.36 | 0.95 |
| Chronic kidney disease^1^ | 2.8 (1) | 13.9 (6) | 5.67 | 0.65-29.55 | 0.11 |
| Immunosuppression ^1^ | 0.0 (0) | 2.3 (1) | N/A |  |  |
| Number of comorbidities^1^ |  |  |  |  |  |
| 0 | 2.·2 (8) | 13.9 (6) | 1.00 |  |  |
| 1 | 33.3 (12) | 34.9 (15) | 1.6 | 0.45-6.13 | 0.44 |
| 2 | 22.2 (8) | 20.9 (9) | 1.5 | 0.36-6.22 | 0.57 |
| 3 | 19.4 (7) | 7.0 (3) | 0.57 | 0.10-3.18 | 0.52 |
| ≥4 | 2.8 (1) | 23.3 (10) | 13.33 | 0.10-3.18 | 0.02 |
| Need for renal replacement therapy^1^ | 8.3 (3) | 67.5 (27) | 22.84 | 5.89-28.52 | <0.001 |
| Time of hospitalization (days)^2^ | 13.9±10.8 | 25.9±12.9 | 1.10 | 1.04-1.16 | <0.001 |

**^1^Values expressed as relative and absolute frequencies.^2^Values expressed as means ± standard deviations. *Information obtained from the patient and/or accompanying members through the initial anamnesis questionnaire during hospital admission.** **OD = Odds ratio; BMI = Body mass index;** **CI = Confidence interval.**

**Supplementary Table 5. Distribution of hospital discharges and in-hospital deaths according to the presence and number of comorbidities in patients hospitalized due to COVID-19 requiring extracorporeal membrane oxygenation from March 1^st^, 2020 to March 31^st^, 2021. Adjusted analysis using logistic regression model (Stepwise method).**

| **Comorbidity*** | **Adjusted univariate analysis** | | | | |
| --- | --- | --- | --- | --- | --- |
|  | **Discharge** | **Death** | **OR** | **95%CI** | **p-value** |
| Need for renal replacement therapy^1^ | 8.3 (3) | 67.5 (27) | 33.93 | 6.79-16.95 | <0.001 |
| Time of hospitalization (days)^2^ | 13.9±10.8 | 25.9±12.9 | 1.08 | 1.02-1.14 | 0.008 |

**Variables associated with in-hospital mortality in patients hospitalized due to COVID-19 between March1^st^, 2020 and March 31^st^, 2021. Analysis adjusted by the logistic regression model (Stepwise methods). Variables included in the final model (stepwise selection) (p<0.2): age, sex, cardiovascular disease, hypertension, diabetes mellitus, number of comorbidities, chronic kidney disease, need for renal replacement therapy and duration of hospitalization. OD = Odds ratio; CI = Confidence interval.**

**Supplementary Table 6.** **In-hospital mortality stratified by age range and number of comorbidities** **in patients hospitalized due to COVID-19 from March 1^st^, 2020 to March 31^st^, 2021.**

| **Age groups (Years)** | **Number of Comorbidities** | | | | |
| --- | --- | --- | --- | --- | --- |
|  | **0** | **1** | **2** | **3** | **≥4** |
| 20-30 | 6 (3.3) | 6 (1.1) | 5 (0.6) | 7 (1.0) | 0 (0.0) |
| 31-40 | 17 (9.4) | 52 (9.5) | 33 (3.9) | 11 (1.5) | 8 (1.1) |
| 41-50 | 29 (16.0) | 67 (12.2) | 55 (6.5) | 34 (4.7) | 24 (3.1) |
| 51-60 | 25 (13.8) | 77 (14.0) | 93 (11.1) | 71 (9.8) | 40 (5.2) |
| 61-70 | 36 (19.9) | 110 (20.1) | 192 (22.9) | 147 (20.4) | 145 (18.9) |
| 71-80 | 29 (16.0) | 92 (16.8) | 200 (23.8) | 193 (26.7) | 231 (30.2) |
| ≥81 | 39 (21.6) | 144 (26.3) | 262 (31.2) | 259 (35.9) | 318 (41.5) |
| Total | 181 (100) | 548 (100) | 840 (100) | 722 (100) | 766 (100) |

**Values expressed as n (%) %. χ2 test: p<0.001.**

**Supplementary Table 7. In-hospital mortality stratified by age range and the need for mechanical ventilation** **in patients hospitalized due to COVID-19 from March 1^st^, 2020 to March 31^st^, 2021.**

| **Age groups (Years)** | **Need for mechanical ventilation** | |
| --- | --- | --- |
|  | **No** | **Yes** |
| 20-30 | 2 (0.5) | 22 (0.8) |
| 31-40 | 9 (2.3) | 112 (4.2) |
| 41-50 | 10 (2.5) | 199 (7.5) |
| 51-60 | 20 (5.1) | 286 (10.7) |
| 61-70 | 40 (10.2) | 590 (22.2) |
| 71-80 | 66 (16.8) | 679 (25.5) |
| ≥81 | 246 (62.6) | 776 (29.1) |
| Total | 393 (100) | 2,664 (100) |

**Values expressed as n(%) %. χ2 test: p<0**.**001.**

**Supplementary Table 8. In-hospital mortality stratified by age range and the need for renal replacement therapy in patients hospitalized due to COVID-19 from March 1^st^, 2020 to March 31^st^, 2021.**

| **Age groups (Years)** | **Need for Renal replacement therapy** | |
| --- | --- | --- |
|  | **No** | **Yes** |
| 20-30 | 11 (0.8) | 7 ( 0.7) |
| 31-40 | 61 (4.2) | 31 (3.2) |
| 41-50 | 107 (7.5) | 64 (6.5) |
| 51-60 | 136 (9.5) | 100 (10.2) |
| 61-70 | 280 (19.5) | 241 (24.6) |
| 71-80 | 299 (20.8) | 291 (29.7) |
| ≥81 | 540 (37.7) | 246 (25.1) |
| Total | 493 (100) | 980 (100) |

**Values expressed as n(%) %. χ2 test: p<0**.**001.**

**Supplementary Table 9 –Comparison of four multivariable logistic regression modelling approaches in combination with goodness of-fit measures (Akaike information criteria and Bayesian Information Criteria).**

| **Characteristics** | **Cox regression model.** | | | | | | | |
| --- | --- | --- | --- | --- | --- | --- | --- | --- |
|  | Model including only age  (Model 1) | | Age adjusted for statistically significant comorbidities and number of comorbidities (Model 2) | | Age adjusted for the need for mechanical ventilation and renal replacement therapy (Model 3) | | Age adjusted for all statistically significant variables  (Model 4) | |
|  | **HR** | **CI (95%)** | **HR** | **CI (95%)** | **HR** | **CI (95%)** | **HR** | **CI (95%)** |
| Age groups (years) |  |  |  |  |  |  |  |  |
| 20-30 | 1·00 |  | 1.00 |  | 1.00 |  | 1.00 |  |
| 31-40 | 1·32 | 0·85-2·06 | 1.29 | 0.83-2.04 | 1.00 | 0.60-1.65 | 1.00 | 0.60-1.06 |
| 41-50 | 1·69 | 1·11-2·59 | 1.65 | 1.08-2.52 | 1.28 | 0.78-2.08 | 1.27 | 0.78-2.07 |
| 51-60 | 2.04 | 1·35-3·10 | 1.90 | 1.25-2.88 | 1.25 | 0.77-2.02 | 1.20 | 0.74-1.96 |
| 61-70 | 3·96 | 2·63-5·96 | 3.41 | 2.26-5.15 | 2.10 | 1.31-3.37 | 1.92 | 1.19-3.09 |
| 71-80 | 5·34 | 3·55-8·03 | 4.32 | 2.86-6.52 | 2.69 | 1.68-4.31 | 2.33 | 1.45-3.76 |
| ≥81 | 7·76 | 5·17-11·65 | 6.39 | 4.23-9.64 | 4.43 | 2.77-7.09 | 3.80 | 2.36-6.11 |
| **AIC** | 49744.70 |  | 49575.29 |  | 35924.56 |  | 35695.62 |  |
| **BIC** | 49830.31 |  | 49703.70 |  | 35992.78 |  | 35729.32 |  |

**HR: Hazard ratio. CI: Confidence intervals. AIC: Akaike Information Criteria. BIC: Bayesian Information Criteria.** **The chosen model was the one with age, comorbidities (Chronic Obstructive Pulmonary Disease, Chronic Kidney Disease, Cardiovascular Disease, Cerebrovascular Disease, Diabetes mellitus and immunosuppression), number of comorbidities, the need for mechanical ventilation and, renal replacement therapy as predictors (lowest AIC and BIC) (Model 4).**

| **Multicentre study cohort** | | | | | | | | | |  |
| --- | --- | --- | --- | --- | --- | --- | --- | --- | --- | --- |
| Authors | Lima et al. | Ranzani et al.^1^ | Kurtz et al.^2^ | Marcolino et al.^3^ | Peres et al.^4^ | De Andrade et al.^5^ | Baqui et al.^6^ | Castro et al.^7^ | Santos et al.^8^ | Zimmermann et al.^9^ |
| Country | Brazil | Brazil | Brazil | Brazil | Brazil | Brazil | Brazil | Brazil | Brazil | Brazil |
| Coverage | Nationwide, private healthcare system | Nationwide, public and private healthcare system* | Nationwide, 126 private ICUs | Nationwide, public and private healthcare system* | Nationwide, public and private healthcare system* | Nationwide, public healthcare system* | Nationwide, public and private healthcare system* | Nationwide, public and private healthcare system* | Nationwide, public and private healthcare system* | Nationwide, public healthcare system* |
| Population | ≥20 years | ≥20 years | ≥20 years | No age restriction | No age restriction | ≥18 years | No age restriction | No age restriction | No age restriction | No age restriction |
| Age (median, IQR or  mean, SD) | 53.2±17.0 | 61 (47-73) | 54 (41-69) | Private: 55 (43-67)  Public: 59 (47-71)  Mixed: 62 (49-74) | 61 (48-73) | 58.9 ± 16.8 | 65.3 ±16.0 (deaths). | 61 (47-73) | Not reported | ≥60 years (52.9%). Median/mean not reported. |
| Hospitalised patients  (n) | 38,937 | 232,036 | 13,301 | 2,054 | 246,025 | 89,405 | 11,321 | 522,167 | 46,285 | 398,063 |
| ICU admissions  (%) | 62.5% | 38.8% | 13,301 | 41.4% | 34% | 22.6% | 35.6% | 33% | Not reported | 26.07% |
| In-hospital mortality (%) | 7.8% | 38% | 13% | 22.2%  Mortality according to hospital type:  Mixed: 26.2%  Public: 24.7%  Private: 10.8% | 37% | 24.4% | 29.4% | 38% | 46.2% | 21.7% |
| Mortality of patients  requiring  mechanical ventilation  (%) | 59.6% | 80% | 59% | 59.5% | Not reported | Not reported | Not reported | Not reported | Not reported | Not reported |

**Supplementary Table 10. Comparison of large COVID-19 cohorts. ***Data originated from the Brazilian Influenza Epidemiological Surveillance Information System, (SIVEP-Gripe) – Available at: <http://plataforma.saude.gov.br/coronavirus/dados-abertos/>.

ICU = Intensive care unit

**Supplementary Table 10. Comparison of large COVID-19 cohorts – Continued.**

| **Multicentre study cohort** | | | | | | | |
| --- | --- | --- | --- | --- | --- | --- | --- |
| Authors | Lima et al. | Docherty et al.^10^ | Grasseli et al.^11^ | Gupta et al.^12^ | Karagiannidis et al.^13^ | ÑamendysSilva et al.^14^ | Jalili et al.^15^ |
| Country | Brazil | United Kingdom | Italy | United States | Germany | Mexico | Iran |
| Coverage | Nationwide, private healthcare system | Nationwide | Lombardy | Nationwide | Nationwide | Nationwide | Nationwide |
| Population | ≥20 years | No age restriction | No age restriction | ≥18 years | ≥18 years | ≥18 years | No age restriction |
| Age (median, IQR or  mean, SD) | 53.2±17.0 | 72·9 (58-82) | 63 (56-69) | 60·5± 14·5 | 72 (57-82) | Not reported | 57.3± 17.6 |
| Hospitalized patients (n) | 38,937 | 20,133 | 3,988 | 2,215 | 10,021 | 131,583 | 23,367 |
| ICU admissions (%) | 62.5% | 14.9% | 3,988 | 2,215 | Not reported | Not reported | Not reported |
| In-hospital mortality (%) | 7.8% | 39% | Not reported | 35.4% | 22% | Not reported | 24.4% |
| Mortality of patients  requiring  mechanical ventilation  (%) | 59.6% | 69% | 51.7% | Not reported | 52.8% | 73.7% | Not reported |

**Supplementary Table 11.** **Proportion of deaths stratified by age range: Comparison of two large Brazilian cohorts (Ranzani^1^ et al vs. Lima et al) of patients hospitalized due to COVID-19.**

| **Age (Years)** | **Proportion of deaths** | **Unadjusted Relative risk (CI 95%)** | **Unadjusted Relative risk reduction (CI 95%)** | **p-value*** |
| --- | --- | --- | --- | --- |
| **20-39** |  |  |  | <0.001 |
| Ranzani et al^1^ | 0.10  (0.106-0.113) | 1.00 |  |  |
| Lima et al | 0.01  (0.011-0.016) | 0.12  (0.10-0.14) | 0.88  (0.85-0.89) |  |
| **40-49** |  |  |  | <0.001 |
| Ranzani et al^1^ | 0.153  (0.150-0.157) | 1.00 |  |  |
| Lima et al | 0.024  (0.021-0.027) | 0.16  (0.137-0.180) | 0.84  (0.81-0.86) |  |
| **50-59** |  |  |  | <0.001 |
| Ranzani et al^1^ | 0.210  (0.210-0.217) | 1.00 |  |  |
| Lima et al | 0.042  (0.037-0.046) | 0.20  (0.17-0.21) | 0.80  (0.78-0.82) |  |
| **60-69** |  |  |  | <0.001 |
| Ranzani et al^1^ | 0.291  (0.290-0.299) | 1.00 |  |  |
| Lima et al | 0.110  (0.106-0.123) | 0.38  (0.35-0.41) | 0.62  (0.58-0.64) |  |
| **70-79** |  |  |  | <0.001 |
| Ranzani et al^1^ | 0.353  (0.349-0.357) | 1.00 |  |  |
| Lima et al | 0.238  (0.223-0.253) | 0.68  (0.63-0.71) | 0.32  (0.28-0.36) |  |
| **≥80** |  |  |  | <0.001 |
| Ranzani et al^1^ | 0.662  (0.657-0.667) | 1.00 |  |  |
| Lima et al | 0.495  (0.473-0.517) | 0.75  (0.71-0.78) | 0.25  (0.21-0.28) |  |

**Upper and lower 95%CI limits were estimated by the Newcombe-Wilson method without continuity correction. * χ2 test.**

**References**

1 Ranzani OT, Bastos LSL, Gelli JGM, et al. Characterisation of the first 250,000 hospital admissions for COVID-19 in Brazil: a retrospective analysis of nationwide data. *Lancet Respir Med* 2021; **9:** 407–418. DOI 10.1016/S2213-2600(20)30560-9

2 Kurtz P, Bastos LSL, Dantas LF, et al. Evolving changes in mortality of 13,301 critically ill adult patients with COVID-19 over 8 months. *Intensive Care Med* 2021; **5:** 538–548. DOI 10.1007/s00134-021-06388-0

3 Marcolino MS, Ziegelmann PK, Souza-Silva MVR, et al; Brazilian COVID-19 Registry Investigators. Clinical characteristics and outcomes of patients hospitalized with COVID-19 in Brazil: Results from the Brazilian COVID-19 registry. *Int J Infect Dis* 2021; **107:** 300–310. DOI: 10.1016/j.ijid.2021.01.019.

4 Peres IT, Bastos LSL, Gelli JGM, et al. Sociodemographic factors associated with COVID-19 in-hospital mortality in Brazil. *Public Health* 2021; **192:** 15–20. DOI 10.1016/j.puhe.2021.01.005.

5 de Andrade CLT, Pereira CCA, Martins M, Lima SML, Portela MC. COVID-19 hospitalizations in Brazil's Unified Health System (SUS). *PLoS One* 2020; **15:** e0243126. DOI 10.1371/journal.pone.0243126.

6 Baqui P, Bica I, Marra V, Ercole A, van der Schaar M. Ethnic and regional variations in hospital mortality from COVID-19 in Brazil: a cross-sectional observational study. *Lancet Glob Health* 2020; **(8):** e1018–e1026. DOI 10.1016/S2214-109X(20)30285-0.

7 Castro MC, Gurzenda S, Macário EM, França GVA. Characteristics, outcomes and risk factors for mortality of 522 167 patients hospitalised with COVID-19 in Brazil: a retrospective cohort study. *BMJ Open* 2021;**11:** e049089. DOI 10.1136/bmjopen-2021-049089.

8 Santos MM, Lucena EES, Lima KC, Brito AAC, Bay MB, Bonfada D. Survival and predictors of deaths of patients hospitalised due to COVID-19 from a retrospective and multicentre cohort study in Brazil. *Epidemiol Infect* 2020; **148:** e198. DOI 10.1017/S0950268820002034.

9 Zimmermann, I.R., et al. Trends in COVID-19 case-fatality rates in Brazilian public hospitals: A longitudinal cohort of 398,063 hospital admissions from 1st March to 3rd October 2020. PLoS One 16:e0254633 (2021). DOI: 10.1371/journal.pone.0254633

10 Docherty AB, Harrison EM, Green CA, et al. Features of 20 133 UK patients in hospital with COVID-19 using the ISARIC WHO Clinical Characterisation Protocol: prospective observational cohort study. *BMJ* 2020; **369:** m1985. DOI 10.1136/bmj.m1985.

11 Grasselli G, Greco M, Zanella A, et al. COVID-19 Lombardy ICU Network. Risk Factors Associated With Mortality Among Patients With COVID-19 in Intensive Care Units in Lombardy, Italy. *JAMA Intern Med* 2020; **180**: 1345–1355. DOI 10.1001/jamainternmed.2020.3539

12 Gupta S, Hayek SS, Wang W, et al. Factors Associated With Death in Critically Ill Patients With Coronavirus Disease 2019 in the US. *JAMA Intern Med* 2020; **180**: 1436–1447. DOI 10.1001/jamainternmed.2020.3596.

13 Karagiannidis C, Mostert C, Hentschker C, et al. Case characteristics, resource use, and outcomes of 10021 patients with COVID-19 admitted to 920 German hospitals: an observational study. *Lancet Respir Med* 2020; **8**: 853–62. DOI 10.1016/S2213-2600(20)30316-7.

14 Ñamendys-Silva SA, Gutiérrez-Villaseñor A, Romero-González JP. Hospital mortality in mechanically ventilated COVID-19 patients in Mexico. *Intensive Care Med* 2020; **46**: 2086–2088. DOI 10.1007/s00134-020-06256-3.

15 Jalili M, Payandemehr P, Saghaei A, Sari HN, Safikhani H, Kolivand P. Characteristics and mortality of hospitalized patients with COVID-19 in Iran: a national retrospective cohort study. *Ann Intern Med* 2020; **174**: 125–127DOI 10.7326/M20-2911

| STROBE Checklist | Item No | Recommendation | Location in Text |
| --- | --- | --- | --- |
| **Title and abstract** | 1 | (*a*) Indicate the study’s design with a commonly used term in the title or the abstract | Page 1 |
|  |  | (*b*) Provide in the abstract an informative and balanced summary of what was done and what was found | Pages 4 |
| Introduction | | |  |
| Background/rationale | 2 | Explain the scientific background and rationale for the investigation being reported | Pages 5-6 |
| Objectives | 3 | State specific objectives, including any pre-specified hypotheses | Page 6 |
| Methods | | |  |
| Study design | 4 | Present key elements of study design early in the paper | Page 6-7 |
| Setting | 5 | Describe the setting, locations, and relevant dates, including periods of recruitment, exposure, follow-up, and data collection | Pages 7-8 |
| Participants | 6 | (*a*) Give the eligibility criteria, and the sources and methods of selection of participants. Describe methods of follow-up | Page 6-8 |
|  |  | (*b*) For matched studies, give matching criteria and number of exposed and unexposed | N/A |
| Variables | 7 | Clearly define all outcomes, exposures, predictors, potential confounders, and effect modifiers. Give diagnostic criteria, if applicable | Page 7 |
| Data sources/ measurement | 8* | For each variable of interest, give sources of data and details of methods of assessment (measurement). Describe comparability of assessment methods if there is more than one group | Page 8-9 |
| Bias | 9 | Describe any efforts to address potential sources of bias | Page 9 |
| Study size | 10 | Explain how the study size was arrived at | Page 7 |
| Quantitative variables | 11 | Explain how quantitative variables were handled in the analyses. If applicable, describe which groupings were chosen and why | N/A |
| Statistical methods | 12 | (*a*) Describe all statistical methods, including those used to control for confounding | Page 8-9 |
| Results | | |  |
| Participants | 13* | (a) Report numbers of individuals at each stage of study—eg numbers potentially eligible, examined for eligibility, confirmed eligible, included in the study, completing follow-up, and analyzed | Figure 1 |
|  |  | (b) Give reasons for non-participation at each stage | N/A |
|  |  | (c) Consider use of a flow diagram | Figure 1 |
| Descriptive data | 14* | (a) Give characteristics of study participants (e.g., demographic, clinical, social) and information on exposures and potential confounders | Page 9-10, Table 1 and 2. |
|  |  | (b) Indicate number of participants with missing data for each variable of interest | N/A |
|  |  | (c) Summarizes follow-up time (eg, average and total amount) | N/A |
| Outcome data | 15* | Report numbers of outcome events or summary measures over time | Page 9-10, Figure 3 |
| Main results | 16 | (*a*) Give unadjusted estimates and, if applicable, confounder-adjusted estimates and their precision (eg, 95% confidence interval). Make clear which confounders were adjusted for and why they were included | Page 9-10, Table 1 and 2. |
|  |  | (*b*) Report category boundaries when continuous variables were categorized | Table 1, 2 |
|  |  | (*c*) If relevant, consider translating estimates of relative risk into absolute risk for a meaningful time period | N/A |
| Other analyses | 17 | Report other analyses done—eg analyses of subgroups and interactions, and sensitivity analyses | N/A |
| Discussion | | |  |
| Key results | 18 | Summarizes key results with reference to study objectives | Page 11-13 |
| Limitations | 19 | Discuss limitations of the study, taking into account sources of potential bias or imprecision. Discuss both direction and magnitude of any potential bias | Page 14-15 |
| Interpretation | 20 | Give a cautious overall interpretation of results considering objectives, limitations, multiplicity of analyses, results from similar studies, and other relevant evidence | Page 14-15 |
| Generalizability | 21 | Discuss the generalizability (external validity) of the study results | Page 14-15 |
| Other information | | |  |
| Funding | 22 | Give the source of funding and the role of the funders for the present study and, if applicable, for the original study on which the present article is based | N/A |
